# The Toxins of Nemertean Worms

**DOI:** 10.3390/toxins11020120

**Published:** 2019-02-15

**Authors:** Ulf Göransson, Erik Jacobsson, Malin Strand, Håkan S. Andersson

**Affiliations:** 1 Pharmacognosy, Department of Medicinal Chemistry, Biomedical Centre, Uppsala University, 75123 Uppsala, Sweden; ulf.goransson@ilk.uu.se (U.G.); erik.jacobsson@ilk.uu.se (E.J.); 2 Swedish Species Information Centre, Swedish University of Agricultural Sciences, 75007 Uppsala, Sweden; malin.strand@slu.se; 3 Linnaeus University Centre for Biomaterials Chemistry, Department of Chemistry and Biomedical Sciences, Linnaeus University, 39231 Kalmar, Sweden

**Keywords:** Anabaseine, cytotoxin, DMXBA, nemertea, nemertide, parborlysin, ribbon worm, tetrodotoxin

## Abstract

Most ribbon worms (phylum: Nemertea) are found in marine environments, where they act as predators and scavengers. They are characterized by an eversible proboscis that is used to hunt for prey and thick mucus covering their skin. Both proboscis and epidermal mucus mediate toxicity to predators and preys. Research into the chemical nature of the substances that render toxicity has not been extensive, but it has nevertheless led to the identification of several compounds of potential medicinal use or for application in biotechnology. This review provides a complete account of the current status of research into nemertean toxins.

## 1. Introduction

**Figure 1:**
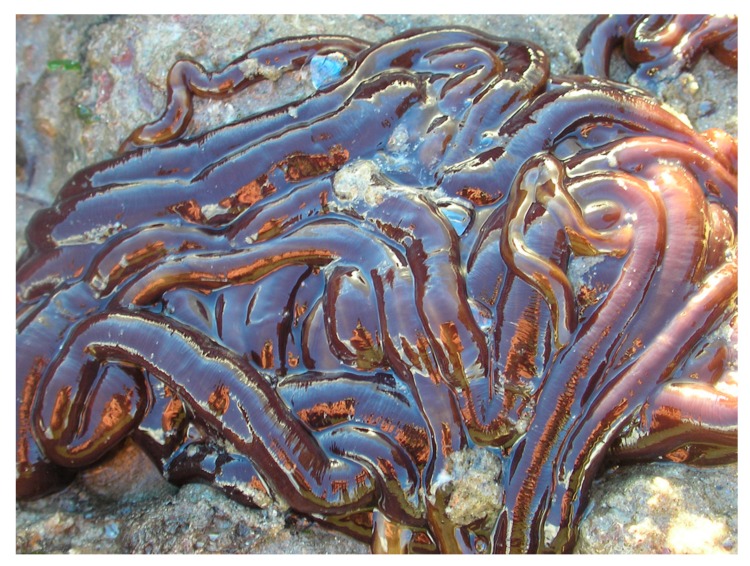
*Lineus longissimus*, the world’s longest animal? Note the characteristic mucus covering the whole body. Photography © Sion Roberts (https://bit.ly/2tlzRAI). Used with permission.

Approximately 1300 species currently comprise the phylum of nemerteans, or ribbon worms (also known as nemertini or rhyncocoeles) [1,2]. Most species are found in marine environments, but 13 terrestrial [3] and 22 freshwater [4] species are described. They are poorly known to the general public and the body of research into nemertean biology and ecology is limited. However, the phylum includes some remarkable species: *Parborlasia corrugatus*, which is the major scavenger on the sea floor in Antarctica, and *Lineus longissimus*, Figure 1, which is known as the longest animal on earth, reaching lengths of 50 m. The eversible proboscis of nemerteans can be armed with a stylet. Certain nemertean species are known to contain remarkably potent toxins: pyridine alkaloids, tetrodotoxin (TTX), and cytolytic or neurotoxic peptides. In the current review, we show the plethora of pharmacologically active compounds that have been discovered in nemerteans.

W.R. Kem, the main pioneer in the area of nemertean chemistry, has previously given detailed accounts [5,6,7,8], but new methodologies and new discoveries prompt a comprehensive update. This review aims to detail the present knowledge on the topic of nemertean toxins with regard to the chemistry, mechanisms of action, and biological functions. Throughout, the term *toxin* is used for any pharmacologically active compound in this paper. We avoid the division into poisons, venoms, or toxungens [9], because delivery mechanisms as well as the potential storage and sources of production of nemertean toxic compounds are only known in parts. In addition, nemerteans are both preys [10] and predators [11], and knowledge is mostly insufficient in determining whether a particular toxin is used for defense or for hunting, or potentially both. The terms nemerteans, nemertean worms, and ribbon worms are used interchangeably.

## 2. Taxonomy and Phylogeny

The classification of nemerteans has been in constant flux, both at the intra-phylum level and with respect to the position of the phylum among the metazoans. Relations between higher taxa are not steadily positioned within a phylogenetic framework and some taxonomic groups within Nemertea are clearly not monophyletic. Traditionally, the two suborders, Anopla and Enopla, have been used following Johnston’s (1837) [12] grouping that is based primarily on the absence or presence of the stylet apparatus in the proboscis. Recently, these suborders were dismissed [13] and instead three classes (natural groups) are maintained from the compiled evidence of the last 15 years: Palaeonemertea, Pilidiophora, and Hoplonemertea. The main morphological features that are used for further classification are muscle layers in the body wall, armament of proboscis (Hoplonemertea), and placement of mouth opening.

Until 2007, 1275 species in 285 genera were counted [2]. This is most certainly an underestimation of the actual number, and genetic evidence (see for example [14]) shows that the sibling and cryptic species are more common than previously recognized.

**Figure 2:**
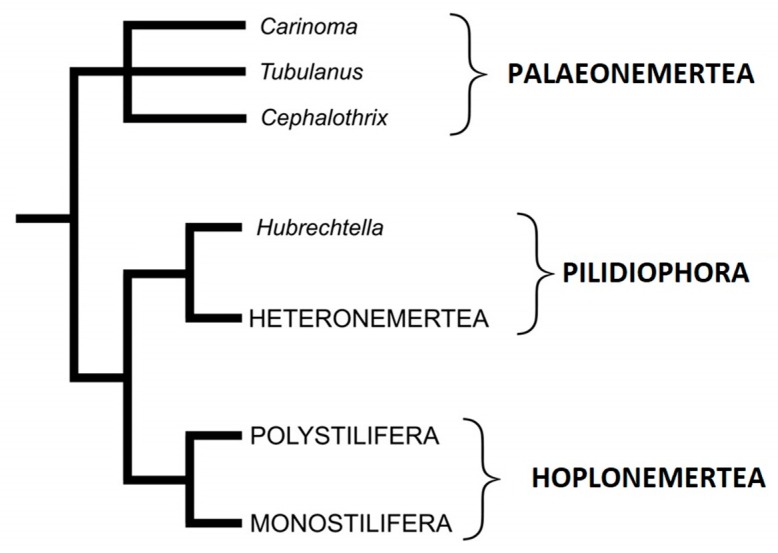
Schematic phylogenetic tree over the Nemertea phylum with names as proposed by Strand et al. [13]. Current intraphylum theories suggest relatively closer relationship between Pilidiophora and Hoplonemertea with Palaeonemertea outside this branch. *Italics* refer to genera.

Phylogenetic analyses support that nemerteans are affiliated to protostome coelomates in Lophotrochozoa. Recent studies support the hypothesis that phoronids (horseshoe worms) are their closest relatives within this group [15]. Intra-phylum phylogeny molecular studies, although based on different markers and non-overlapping taxa, have agreed at some fundamental points: monophyly of Hoplonemertea, paraphyly of Anopla [16]. The proposed modified taxonomic structure is hence presented in Figure 2. 

## 3. Anatomy

**Figure 3:**
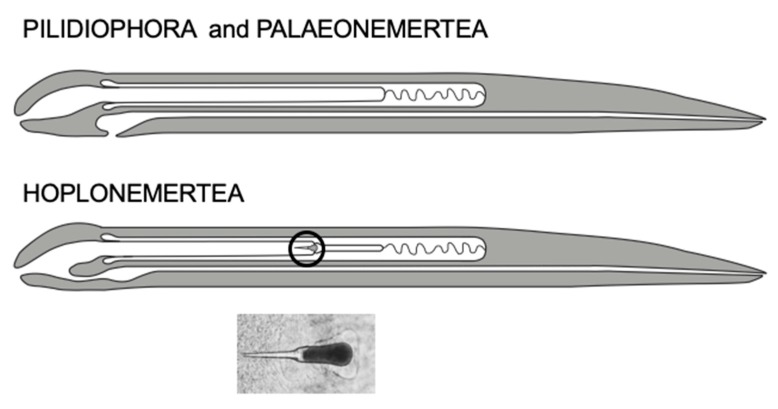
Differences between the Pilidiophora and Hoplonemertea anatomies. **Top**: The mouth opening of Pilidiophora and Palaeonemertea is separated from the proboscis, whereas a common opening is the case for Hoplonemerteans. **Bottom**: The hoplonemertean proboscis (in circle) contains a stylet (or several in the case of *Polystilifera*) by which a prey epithelium can be punctured. Photography of a stylet from *Amphiporus lactifloreus*.

Nemerteans are unsegmented animals with an eversible proboscis and the capability of extreme contraction/elongation as distinctive features. Many species are brightly coloured with different patterns of pigmentation. Different species range in size from microscopic, such as *Carcinonemertes* sp., which only reaches 2 mm in length, to 50 m, as reported for *Lineus longissimus*. The most prominent synapomorphic anatomical feature of nemerteans is the eversible proboscis that lies within a special coelom called the rhynchocoel. A well-developed nervous system renders the ability to detect and catch prey with precision. The proboscis is used to catch prey (e.g., molluscs, crustaceans, worms) and the toxin(s) are is considered to be concentrated to the anterior part of the proboscis [17]. The hoplonemerteans carry a stylet [18], Figure 3, that is attached at its outermost tip, and in association a sac assumed to contain toxins [19]. This armed proboscis can puncture the prey and quickly immobilize it. The nemertean can then feed upon the prey.

Nemerteans that do not belong to the hoplonemerteans generally lack the stylet but they can still be very effective predators. Certain cells form papillous structures on the proboscis [20,21], releasing glandulous secretions that may contain both toxins and glue-like substances that help to hold the prey until death/immobilization occurs [22]. Tissue dissolving capacities seem to have been exhibited by some of these substances. Among these structures, the so-called pseudocnidae have been subjected to detailed study [23,24], but more work remains in order to ascertain their role in toxin production and delivery.

Most species secrete epidermal mucus that covers the whole body. It facilitates the “gliding” movement of the animals. Many species respond to tactile stimuli with enhanced mucus production. This mucus is not well chemically explored, although some studies have displayed the presence of both cytolytic toxins and neurotoxins [8,25,26]. The mucoid neurotoxins could be functional in chemical defense or pheromonal/odour-driven activities (chemical signalling), but there is little empirical evidence in the literature.

## 4. Early Descriptions of Ribbon Worms and Their Toxins

**Figure 4:**
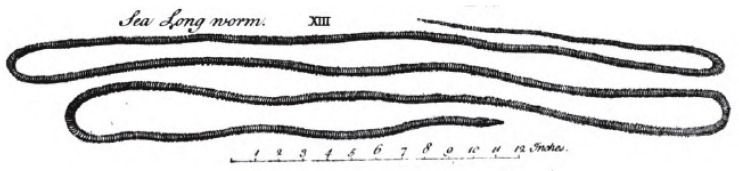
The “Sea long worm”, an early depiction of what is likely *Lineus* sp. Reproduced from [29], 1758, Oxford.

The perhaps earliest description of a ribbon worm in the literature is also the first indication of its toxicity. The Swedish ecclesiastic Olaus Magnus described in his *Historia de Gentibus Septentrionalibus* from 1555 [27] how a (*worm is entirely harmless, unless touched by a human hand. In that case, the fingers will swell when the animal comes into contact with the skin of the hand*). The credibility of the source may be somewhat hampered by the fact that it also contains descriptions of giants, sea monsters, and unicorns, but the species discussed has nevertheless been interpreted as *Lineus longissimus* [28], Figure 1. An early illustration of a ribbon worm (possibly *L. longissimus*) is seen in William Borlase’s *Natural History of Cornwall* [29], Figure 4. It was denoted a “Sea long worm” and then categorized as belonging to the “less perfect kind of sea-animals”, but no mention was made of any toxicity.

**Figure 5:**
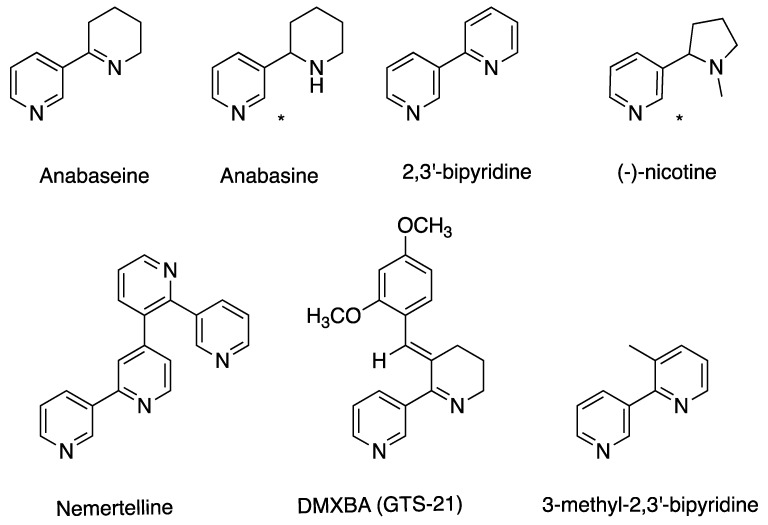
Structures of nemertean pyridine alkaloids, nicotine, and the derivative 3-(2,4-dimethoxybenzylidene)-anabaseine (DMXBA).

In 1900, Wilson described the toxicity of the mucus as “*it will be found so intensely arid as to parch the whole mouth, and the taste remains for a long time*” after having placed a drop of mucus from the heteronemertean *Cerebratulus lacteus* on the tongue [30]. Reisinger [31] described the attack of *Prostoma graecense* and then suggested that the paralyzing poison originated from the epithelium of the posterior proboscis. The first systematic investigations of the toxic matter were carried out in the 1930’s by Bacq [32,33]. An account was given by Kem [5], which, in brief, is described here: aqueous extracts of whole hoplonemerteans (*Amphiporus lactifloreus* and *Drepanophorus crassus*) were shown to exhibit a nicotine-like effect on frog smooth muscles. The *A. lactifloreus* extract was still active after boiling under acid and alkaline conditions both, and the active compound could be extracted into chloroform under basic conditions. Bacq named this substance “amphiporine” and then concluded that it was an alkaloid similar to nicotine, Figure 5. The nicotine-like effect appeared to be absent in heteronemerteans, but extracts of those were shown to induce repetitive spiking in an isolated crab nerve preparation. As this effect appeared to be general for nemerteans, it was assumed that the effect came from another type of toxin, which was named “nemertine”. In 1939, King [34] reported a series of attempts to purify the amphiporine fraction, but was unable to decipher the identity of this toxin, and since then it appears that no further studies were reported regarding the nature of nemertean toxins until the early 1970’s.

## 5. Ribbon Worm Toxins

The discovery of ribbon worm toxins on a molecular level can be attributed to a few research groups, with each focusing on their specific types of target compounds. Kem and co-workers pioneered pyridine alkaloids. TTX and analogs have been the focus of research groups from Hiroshima and Tokyo, and also lately Vladivostok. Peptide toxins have been explored by the groups of Kem and Blumenthal. In recent years, the interest in this field has been reinvigorated by the development of techniques, such as peptidomics and next-generation sequencing. In addition to the known toxins, gene sequences of other peptides and proteins that are related to toxins known from other organisms have been identified. These sequences represent a fourth category, albeit the presence of the actual molecules have not yet been confirmed. This review follows this subdivision.

### 5.1. Pyridine Alkaloids

During the summer of 1967, W.R. Kem collected some 10,000 specimens of Paranemertes peregrina along the coast of San Juan Island, aiming to uncover the identity of Bacq’s amphiporine [33]. The nemerteans were homogenized, centrifuged, and then subsequently extracted with chloroform. The effects of resultant extracts were studied in bioassays of crabs (*Hemigrapsus nudus*) and crayfish (*Cambarus virilis*). A toxic fraction was obtained and then subjected to mass spectrometry (MS) and NMR analyses, identifying the active principle as anabaseine [35], Figure 5. This was followed by an extensive investigation of the localization of anabaseine in *P. peregrina*, and its occurrence in other nemertean species [17]. The concentration of anabaseine was found to be the highest by far in the proboscis, where it is concentrated to the anterior and median regions. High concentrations were also found in the peripheral part of the body wall. In addition to *P. peregrina*, anabaseine was present in the stylet-carrying *Amphiporus lactifloreus* and *Tetrastemma worki*, whereas it was not present in any of the other species that were studied. Kem et al. identified two major alkaloids in *A. angulatus*, neurotoxic 2,3′-bipyridyl and a tetrapyridyl, they named nemertelline because of its similarity with the tobacco alkaloid nicotelline [36], Figure 5. Later, the structure of nemertelline was revised by Cruskie et al. [37]).

In another study, the content of pyridine alkaloids in 19 nemertean species was assayed [38]. The analysis was complicated by limited sample availability of some species and long storage times, resulting in the possible degradation of toxic constituents. Nevertheless, it was shown that anabasine (not to be confused with anabaseine), Figure 4, was present in two of the species, *Amphiporus angulatus* and *Zygonemertes virescens* [38]. Moreover, several other unidentified pyridines appeared to be widespread among the species that were analyzed. A later study showed the presence of 15 different alkaloids in a basic chloroform soluble fraction derived from *A. angulatus* [39]. However, the details of the analysis and the identities of the alkaloids were not described. One of the compounds found in *A. angulatus*, 3-methyl-2,3′bipyridyl, was later identified and synthesized [40].

The pyridyl compounds that were identified were all shown to be active against invertebrates in the μM range, Table 1. A series of bioassays were carried out for the whole range of compounds and synthetic derivatives: crayfish paralysis, feeding behaviour of spiny lobsters, electrophysiological recordings of chemoreceptor neurons, and patch clamp recordings of crayfish gastric mill chloride channels. The results suggested that crayfish paralysis was due to the effect of pyridyl toxins on nicotinic cholinergic receptors in the crustacean central nervous system.

The results also indicated the partial deterrence of spiny lobsters from *A. angulatus* toxins. Cleaning off the mucus from the nemertean led them to be consumed. In addition, nemertelline (inactive in the other assays), together with anabaseine and 2,3′-bipyridyl, stimulated a stomatogastric muscle nicotinic receptor calcium channel of crayfish. Permanent cation derivatives of 2,3′-bipyridyl maintained activity on this receptor, a result that is in contrast to the case for CNS activity, supposedly due to the permanent charge hindering blood-brain passage. These results, taken altogether, suggested that the various pyridine compounds may have different activities and that their combined presence brings on a multimodal defense [39]. 

A later study showed that 2,3′-bipyridyl, anabaseine, nemertelline, and anabasine all inhibited barnacle larvae settlement. Analysis of effects from a series of analogues to the natural pyridyl alkaloids suggested that both of the nitrogen atoms of 2,3′-bipyridyl are important for action, as are their relative positions. Moreover, the protonation of these nitrogens inhibited the antifouling activity [41]. This work generated a patent covering application of these and related compounds as anti-fouling materials [42]. To our knowledge, these compounds have not been developed further for this purpose.

In the 1970’s, Kem observed that the toxicity of anabaseine was not exclusive to crayfish, and that it was equally potent as nicotine when injected into mice [36]. This prompted studies of its potential pharmacological activities. Meyer et al. demonstrated that anabaseine stimulated acetylcholine release from the rat brain cortex [43], and anabaseine activity was subsequently studied in comparison to a series of alkaloids, including nicotine and anabasine to vertebrate nicotinergic receptors [44]. It was shown that anabaseine selectively stimulates nicotinic receptors, in particular, α-7 and neuromuscular type receptors.

**Figure 6:**
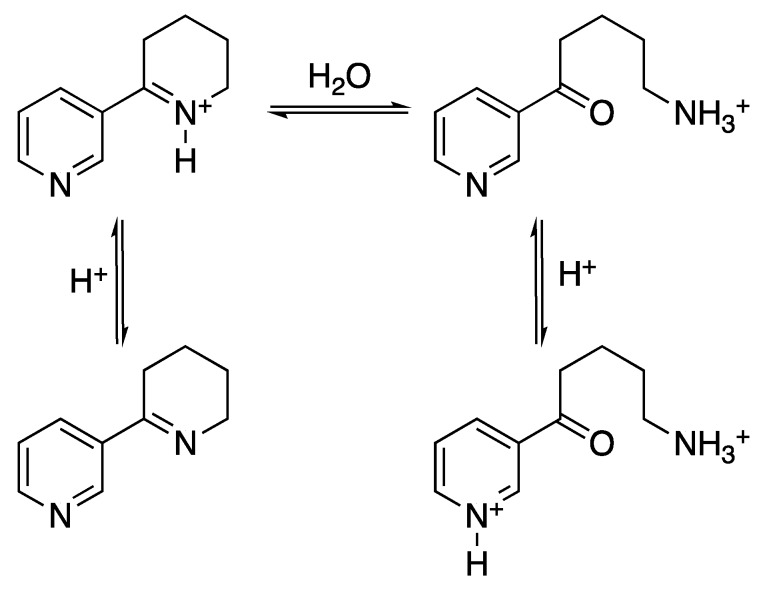
Anabaseine equilibrium states. Lower pH shifts the equilibrium towards the hydrolyzed product, whereas the cyclic anabaseine is favoured by higher pH. Adapted from [45].

The comparative analyses provided an understanding of some structural aspects of anabaseine recognition. Moreover, a systematic study of the anabaseine solution equilibrium demonstrated how its molecular structure shifts with pH. At neutral pH, it exists in an equilibrium with an almost equal amount of protonated cyclic structure and the corresponding hydrolyzed product and minor amounts of the non-protonated cyclic species, the abundance of which increases with pH [45], Figure 6. 

Taken together, this provided rationale in understanding the effects of a series of cinnamylidene and benzylidene derivative of anabasine, among them 3-(2,4-dimethoxybenzylidene)-anabaseine or DMXBA [46], also known as GTS-21 [47]. It is the anabaseine derivative that has come closest to medicinal application. DMXBA shows long-term potentiation via the selective binding to CNS α-7 nicotinic acetylcholine receptors [47]. This sparked interest by the similarity to effects of nicotine on memory-related behaviours, specifically Alzheimer’s disease [48]. DMBXA showed promising results in eyeblink classical conditioning (EBCC), a rabbit model to parallel aging in humans [49]. It was also shown to improve memory-related behaviour in Sprague–Dawley rats [50]. DMXBA in Alzheimer’s has been reviewed by Zawieja et al. [51], and Kem et al. reviewed studies on the use of anabaseine and DMXBA against memory loss and schizophrenia [46]. A comprehensive paper by Rangel and Falkenberg discussed the recent status of clinical trials with regard to compounds of marine origin and their derivatives in the pharmaceutical pipeline [52]. As of January 2019, eight studies regarding DMXBA are listed in ClinicalTrials.gov, but no outcome is reported.

An overview of nemertean species (and their place of origin), which have been analyzed with respect to anabaseine related compounds, is presented in Table 2.

### 5.2. Tetrodotoxin (TTX)

**Figure 7:**
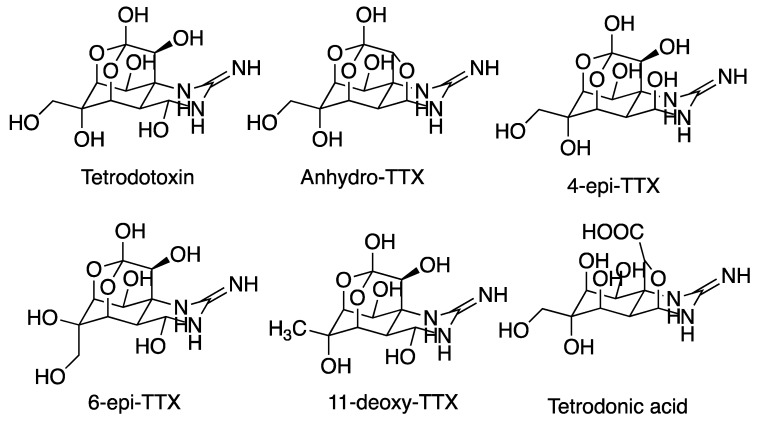
Tetrodotoxin (TTX) and a selection of active analogs identified in nemertans.

TTX, as in Figure 7, is well known as the toxin in puffer fish [55], but it has been found in several other genera, including salamanders, frogs, octopi, flatworms, and crustaceans [56]. It acts via binding to the extracellular pore opening site 1 (*i.e.* the P-loop between domain V and VI) in voltage-gated sodium channels (VGSC). Thereby, it blocks Na^+^ conduction and causes its strong paralytic effect [57]. TTX has been a key tool for the characterization of ion channels and for fundamental studies in neurology as a selective blocker [58], and it is still widely used as a pharmacological tool. In addition, it is the subject of several clinical trials for its potential use in pain relief [59,60]. The biosynthesis of TTX is not clear, but bacterial and/or symbiotic routes have been suggested [61]. However, there are no methods to produce TTX in sustained cultures [62] and TTX production is not feasible for commercial synthesis [56]. TTX production therefore relies on purification from pufferfish; 1–2 g of TTX is considered to be a good yield from 100 kg of puffer fish ovaries [63].

Miyazawa et al. were the first to report the occurrence of TTX in ribbon worms [64], namely in *Lineus fuscoviridis* and *Tubulanus punctatus* that were collected from intertidal zones in the south of Japan. TLC showed the presence of TTX, which was confirmed by HPLC, GC-MS, and lethal effects on mice measured in mouse units (MU). One (1) MU corresponds to the death of one mouse within 30 min. Out of 56 sampled individuals (both species), 32 were found to contain toxin in the range 10–500 MU/g live nemertean.

The same group showed that *Cephalotrix linearis* was about tenfold more active [65]. Toxicity was again ascribed to TTX, and the compounds were suggested to be localized to the proboscis, as this exhibited about twice the lethal potency (MU/g) as the rest of the body. The potency of mucus was about ¼ of that of the proboscis. In retrospect, other compounds may have contributed to these results. A concurrent study suggested that one unknown such toxin, called “tetrodonic acid-like substance” was a likely precursor to TTX, although no structures were characterized [66]. A few years later, Asawaka et al. conducted surveillance work by the Hiroshima Bay oyster farms for sources to paralytic shellfish poisoning (PSP). They found that “himomushi” (*Cephalotrix* sp.), clinging on to oyster shells, showed paralytic toxicity that could be attributed to TTX and its derivatives [67], as identified by HPLC and GC-MS. Although the toxic content substantially varied among the worms that were collected, the potency was noteworthy, the most active sample exhibiting 14,734 MU/g. This prompted an extensive investigation into the toxic nature of the himomushi toxin. LC-MS and NMR evidence conclusively demonstrated that the observed activity was due to TTX [68].

A more recent paper detailed toxicological surveillance work carried out between 1998–2005 at several Japanese sites. Out of 764 specimens of *Cephalothrix simula* that were collected, approximately 80% exhibited strong toxicity (≥1000 MU/g), which was ascribed to TTX and the derivatives 4-epi-TTX and 4,9-anhydro-TTX [69]. This suggests that TTX is a common constituent of *C. simula*, at least in these waters. In addition, HPLC and GC data indicated that TTX was also present in other species, e.g., *Lineus torquatus*, *L. alborostratus*, and *Nipponemertes punctatula*.

TTX does not appear to be synthesized by the nemerteans themselves, so how does this toxin emerge in these worms? The possibility of bioaccumulation, as has been observed e.g., in *Fugu* from eating TTX-containing flatworms [70], is one possibility. However, a common hypothesis suggests that the presence of TTX is due to production from commensal bacteria. *Vibrio alginolyticus* bacteria was provisionally identified in the intestinal contents as a plausible source of TTX. Previous work by Carroll et al. [71,72] had elaborated on the idea that a symbiotic relationship between *Vibrio* bacteria and nemerteans might be the origin of TTX. *V. alginolyticus* was isolated from the epidermal mucus and whole body extracts of a selection of nemertean species that were collected outside of North Wales. The extracts and bacteria cultures that were grown in the presence of the nemertea samples were then subjected to ultraviolet UV spectroscopy, from which it was concluded that TTX was present in several of these samples. At a closer look, this data is inconclusive at best. Neither did HPLC analyses, where the samples were compared to puffer fish TTX controls, provide solid evidence for the presence of TTX, because the retention time of the “TTX” differed between the samples.

Nevertheless, it inspired an idea to use *V. alginolyticus* cultures that were grown with mucus from *L. longissimus* as a continuous production system for TTX [73]. Although toxicity was evident, as assayed using *Carcinus maenas* (green crab), an in-depth analysis of this system was unable to identify any traces of TTX. Moreover, the toxic action was found to originate from the mucus, and it was unrelated to the presence of *V. alginolyticus* [25]. This work highlights the difficulty of conclusively demonstrating the presence of the complex TTX molecule, and it is not the first time that doubts have been raised. In two papers, [74,75], Matsumura challenged claims of TTX production in *Vibrio* sp. cultures. Two standard methods were employed: HPLC-UV and GC-MS. High “TTX” peaks were found by both techniques, but these peaks were also found in the polypeptone and yeast extracts that were used for cultivation. It is obvious that the sole use of HPLC-UV may generate false TTX positives. In addition, the common GC-MS method is based on harsh alkali hydrolysis, leading to a C9 base compound, which is common to a group of related compounds. 

The need for caution can further be stressed as judged by a study by Salvitti et al. [76]. They reported the analysis of 102 bacterial strains that were isolated from the marine slug *Pleurobrancheaea maculata* and the marine flatworm *Stylochoplana* sp., whereby both have previously been shown to contain TTX [77], without finding any evidence for TTX in these cultures [76]. A literature survey in that paper of 25 reports on bacterial TTX production showed that they relied on indirect evidence in all cases but one, in which HPLC-MS data was included. However, there is also support for the hypothesis that bacteria produce TTX with or in conjunction with nemerteans. Beleneva et al. isolated bacterial strains from *Cephalothrix simula* and then identified TTX producing cultures using polyclonal rabbit TTX-antibodies, Alexa 488 secondary antibodies, and confocal microscopy [78]. A positive result was found for *Bacillus* sp. 1839. Transmission-electron microscopy was then used to localize antibody binding, both to immature forespores and to mature spores of the bacteria [79]. Lately, the screening of total bacterial cultures that were isolated from a number of nemertea species indicated TTX-positive cells originating from *Hubrechtiella juliae* and *Lineus alborostratus* [80]. A previous study had used anti-TTX monoclonal antibodies to identify TTX at several epithelial and intestinal sites in *Cephalotrix* sp. [81], and recently *Lineus alborostratus* was subjected to a similar analysis, generating detailed pictures, which indicated that TTX was primarily located to the cutis layer, and a hypothesis for its intracellular delivery was also proposed [82]. A 2018 study by Vlasenko et al. [83] provided HPLC-MS/MS evidence regarding the presence of seven different TTX analogues in extracts from *Cephalotrix simula* and three in *Kulikovia manchenkoi*. However, the presence of TTX itself was not observed. In another study, Kwon et al. combined MALDI-MS with cytotoxicity assays on HPLC fractions from *Yininemertes pratensis*, which were collected in the Han River estuary in South Korea. Several masses corresponding to known TTX derivatives were found, as was cytotoxicity in certain fractions. However, correlation between the two was poor, again raising the question of the origin of activity [84].

To conclude, the evidence that TTX is present in a range of nemertean species is accumulating, especially for *Cephalotrix* sp. that were collected in Japanese/Russian waters, but many observations rely on the selectivity of TTX antibodies. It is not clear to the authors of this review that the risk of cross reactions can be completely excluded. Nearly all of the examples where nemertean worms have been shown unequivocally to contain TTX originate from the Sea of Japan or its vicinity, although a recent report of *Cephalothrix simula* caught in Cornwall, UK, demonstrated TTX and TTX derivative content analyzed by HPLC-MS/MS [85]. Although originating from the Pacific, this species appears to be establishing itself in Europe [86], which is why this first observation in the UK was not totally unexpected.

The apparent geographical concentration of TTX bearing nemerteans could of course be a function of limited search efforts elsewhere, but it does raise questions regarding the general role of TTX versus other known toxins, such as peptides, anabaseine, and other pyridyl compounds. An overview of nemertean species that were analyzed with respect to TTX related compounds is presented in Table 3.

### 5.3. Peptide Toxins

#### 5.3.1. Cerebratulus Toxins

##### B-neurotoxins

Some advances into the nature of Bacq’s nemertine were reported already in the early 1970’s [5,17]. This will be touched upon in Section 5.3.3. However, to shed further light on the identity and the character of nemertine, Kem turned to *Cerebratulus lacteus*, extracting mucus from 160 specimens, which were then subjected to several rounds of purification [89]. Two fractions that were obtained by size exclusion chromatography stood out with regard to their toxic activity, the first containing several compounds of of approximately 11 kDa. These were designated *Cerebratulus* A-toxins. The A-toxin fraction was toxic to mice as well as crayfish, but its toxicity appeared to be more gradual and paralysis was less severe than in the case of a second fraction, containing smaller proteins, designated *Cerebratulus* B-toxins. The B-toxins were highly toxic to crayfish, leading to convulsions, persistent paralysis, and death, but they affected neither cockroach nor mice. Several steps of CM-cellulose gradient purification led to the identification of four main proteins: B-I to B-IV. It was hypothesized that the four proteins are homologous, with molecular weights of approximately 5.4 kD (B-I) and 5.9 kD (B-II to IV), containing six (B-I) or eight (B-II to IV) cysteines. Although B-II appeared to be the most toxic compound, when assayed against *Procambarus clarkii*, more focus was placed on B-IV, on account of availability (B-I: 7.0 mg; B-II: 6.0 mg; B-III: 3.9 mg; B-IV: 101.0 mg). The full primary sequence of B-IV was resolved the same year by Blumenthal and Kem [90] (later revised, [91]). Subsequently, the primary sequence of B-II was reported, demonstrating a high degree of homology between the two peptides (41 out of 55 amino acids were identical); both contained a hydroxyproline at position 10 and four disulfides [91]. In parallel, the structural features of relevance for B-IV activity were identified. It was shown that the nitration of tyrosine-9 almost completely abolished the toxic activity on *P. clarkii* without inducing any major disruptions to the secondary structure [92], and via reaction with 2-hydroxy-5-nitrobenzyl bromide (HNB), it was shown that Trp-30 was similarly crucial for activity, whereas Trp-5 was apparently not [93]. Reduction of the disulfides generated a structure that was devoid of *P. clarkii* activity, whereas renaturation restored activity [94].

In attempts to identify the target receptor, Toth and Blumenthal used sucrose gradient centrifugation to purify membrane fractions of lobster (*Homarus americus*) nervous tissue [95]. The axonal vesicles that were obtained were found to bind B-IV with a single class of binding sites at a K_d_ of 5–20 nM, and a binding capacity of 6–9 pmol/mg membrane protein. The pharmacological activity of B-IV is consistent with the effects on ion channels in the nerve membrane, and from competition experiments with other toxins and studies of Na^+^ flux, it was suggested that action was most likely due to effects on voltage-gated Na^+^ channels or K^+^ channels. In a follow-up study, a pull-down strategy was attempted, where B-IV was conjugated with ^125^I-azidosalicylic acid (ASA), followed by the incubation of this derivative with lobster axonal and muscular vesicles, respectively. The derivative was thereby linked photochemically to the receptor. After subsequent SDS-electrophoresis, major bands could be identified for both preparations, which, after correction (B-IV, ^125^I-ASA masses subtracted), gave the molecular masses 40 and 38 kDa, matching those of ß_1_ and ß_2_ subunits of mammalian Na-channels. Accordingly, the authors tentatively suggested that binding occurred at these components of the Na^+^ channel in lobster nerves [96].

**Figure 8:**
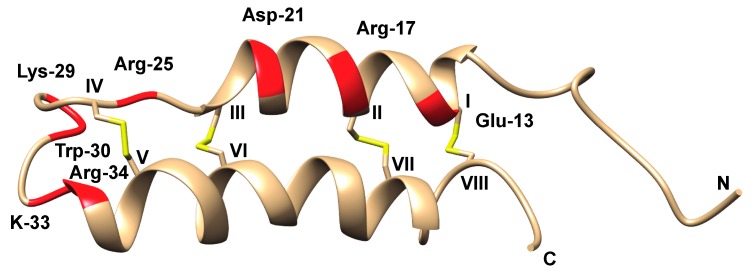
B-IV NMR structure (RCSB id: 1VIB) [99] with residues important for activity marked in red, Cys residues in roman numerals, and disulfide bonds in yellow.

Detailed investigations of the three-dimensional (3D) structure of B-IV have been carried out. Using circular dichroism (CD) and Raman spectroscopy, Kem et al. showed that B-IV had a high α-helix content (49–78%), but no detectable ß-sheets [97]. This character was subsequently confirmed by ^1^H-NMR, showing the presence of two α-helices, incorporating residues 13–26 and 33–49, and a helix-like structure was also seen in the five C-terminal residues, whereas the N-terminus appeared to be unordered [98]. In 1997, Barnham et al. demonstrated the well-defined helical hairpin structure that was obtained in solution via 600 MHz ^1^H-NMR, Figure 8 [99]. 

Recombinant production was first reported by Howell and Blumenthal in 1989 [100]. A synthetic B-IV gene was introduced via a plasmid in *E. coli* and produced as a fusion protein either with ß-galactosidase or the gene 9 protein of bacteriophage T7, which was subsequently cleaved off. The peptide folded as natural B-IV, differing only by virtue of an extra methionine at the N-terminus and the replacement of Hyp-10 for Pro, with specific toxicity of 35-40% to that of the naturally occurring form. The extra codon for Met was deleted in the following study [101], and the resultant peptide was shown to be fully active. Using the recombinant approach, a series of mutants of the Met-B-IV was produced, where N-terminal alanines (3 and 8) were replaced by serines or glycines. Interestingly, the double-Ser mutant was about two-fold more active than its parental form, whereas the double-Gly mutant was slightly less active. No effects on folding were observed from these mutations, as analyzed by CD. In two follow-up papers [102,103], a series of recombinant mutants were compared. Table 4 summarizes the outcomes of these mutations, as well as those from earlier studies. 

The extensive study of the B-IV neurotoxin by Blumenthal and co-workers has provided a detailed understanding of structural features that are crucial for activity. Arg-17 is a key amino acid for activity, lost upon replacement by either Gln, Ala, or Lys. Likewise, when Arg-25 is replaced by Gln, activity is lost, whereas replacement by Lys does not alter activity. At position 30, Trp can be replaced by other aromatic amino acids without the loss of activity, whereas replacement by Ser leads to a 40-fold reduction. A number of other positively charged amino acids are of importance for activity, albeit less so than those mentioned above [102], Figure 8.

##### Cerebratulus A-Toxins

The larger (11 kDa) *Cerebratulus* A-toxins have also been rigorously studied, and progress has been thoroughly reviewed [26]. Briefly, initial experiments [89] were carried out on a size exclusion chromatography (SEC) fraction rather than on purified toxin, but already at this stage, it was clear that the A-toxins exhibited a cell-lytic mode of action. In a following study, partial (A-I) and full purification (A-II to A-IV) was achieved, and the peptides were subjected to both structure and activity studies [104]. The N-termini of the latter three were sequenced by Edman degradation, showing chemical homology (seven positions of 12 were identical) and amino acid analysis revealed very high Lys content, and the presence of three (A-II, A-III) or four (A-IV) disulfides. In one study, A-IV was shown to inhibit phospholipid-sensitive Ca^2+^-dependent protein kinases [105], but, beyond this, most of the subsequent effort has focused on A-III: its full primary sequence was determined [106], as was its disulfide connectivity [107]. A high α-helical content was suggested from these studies, and Dumont and Blumenthal studied this peptide using CD [108], indicating that A-III contained 37% α-helix and 14% ß-sheet structure. An enzymatic cleavage product (1–86) exhibited four-fold lower cytolytic effect as compared to the native peptide, suggesting that the proposed helical sequence (63–95 was suggested to consist of an amphipathic helix) of the C-terminal is important for the lytic activity. This activity was known already from the first paper while using purified toxin [104], where a number of assays were carried out, the most striking result of which being the hemolytic activity on human erythrocytes (A-III: HC_50_ 0.3 μmol/L). LD_50_ measurements of A-II–A-IV were performed on crayfish and fiddler crab (values ranging between 0.1–0.5 mg/kg) and mouse (1.5–2.8 mg/kg). In addition, paralytic and hemolytic activity studies on extracts from different body parts indicated that the A-toxins are mainly present in the nemertean integument. In another study, the cardiac effects of A-III toxin were investigated on canine cardiac Purkinje fibers [109]. Brief exposure of sublytic concentrations led to reversible membrane depolarization, but the addition ofCa^2+^ was found to reduce this effect. Higher concentrations (>2 mg/L) led to irreversible cell damage.

As for the underlying explanation for cell lysis, a number of studies were carried out. A-III-liposome experiments indicated that the N-terminal end plays an important role for the insertion of the peptide into the membrane [110]. This was followed through with release studies on DOPC-containing liposomes showing an A-III concentration dependent release of small molecular markers [111]. It was shown that tetrameric concanavalin-A leaked out from the liposome following the addition of A-III, indicating that the lesion produced exceeded 90 Å, corresponding to the rotational diameter of Con-A. Blumenthal and co-workers investigated the mechanism of A-III hemolysis in a series of papers [112,113,114,115], which showed that A-III forms tetramers at the membrane, a formation that is promoted by oleic acid binding at specific sites of the peptides. High concentrations of mono- and divalent ions were seen to inhibit hemolysis, by analogy to the Posner and Kem study [109], proposedly due to the inhibition of A-III self-association following membrane binding.

#### 5.3.2. *Parborlasia* Toxins

In 1991, Heine et al. reported a multi-faceted investigation on *Parborlasia corrugatus*, a top predator and scavenger in the Antarctic fauna [116]. They demonstrated cytotoxicity of whole body extracts (aqueous, 3%), which proved to be lethal to spermatozoa of the Antarctic sea urchin *Sterechinus neumayeri.* Moreover, feeding experiments demonstrated significant deterrence both for the Antarctic cod (*Dissosticus mawsoni*) and for the benthic fish *Trematomus bernacchi*. The identity or type of toxins involved was not investigated further, although a later review stated that the worm harboured a “potent toxic neuropeptide” [117], which is an early hint of ongoing work where the freeze-dried mucus from *P. corrugatus* was also analyzed by chemical means. This work [118] involved the size exclusion of resuspended lyophilized mucus, followed by HPLC fractionation on a hydroxyapatite column. Hemolytic fractions were further chromagraphed on C18 and C4 using MeCN/H_2_O gradients. One fraction displayed hemolytic activity towards erythrocytes, and it was subject to electrophoretic analysis, and subsequent MALDI-TOF analysis revealed the presence of two ions, one at 10,324 Da and a less prominent at 10,097 Da. N-terminal Edman sequencing provided a partial N-terminal sequence, which also suggested the presence of isotoxins, but the authors were unable to separate these compounds. Sequence alignment of the first 25 amino acids of the protein, named parborlysin, to the *C. lacteaus* A-III cytolysin demonstrated 70% homology. The pI, 9.1, also pointed to a close relationship between these proteins. However, some differences were evident; (i) erythrocyte lysis via *Cerebratulus* A-toxins is Ca^2+^ sensitive, but parborlysin was adversely affected by Ca^2+^; (ii) the identities and concentrations of the added membrane lipids required the inhibition of A-III and paborlysin lysis, respectively, varied substantially. Twelve years later, a full length parborlysin sequence was reported by Butala et al. [119]. Furthermore, by PCR amplification and sequencing, six additional parborlysins were identified, whereby all but one contained six cysteines, suggesting three disulfides in the structure. The peptides were all in the range 9400–10,100 Da, with a calculated pI in the interval 10–10.4. A putative structure was modelled *ab initio* from the sequence, suggesting a globular peptide with secondary structure that solely consists of alpha-helices. Parborlysin sequences were also cloned into expression vectors that were introduced into *E. coli* and then subsequently expressed. The process was complicated by the inherent toxicity of parborlysin, and although it was shown that the construct had indeed been produced in one case, no hemolytic activity could be shown. This suggested that the protein was not properly folded, or that the N-terminal peptide requires a free N-terminal end for activity, which was blocked by a His-tag.

#### 5.3.3. *Lineus* Toxins

In a 1971 paper, Kem reported the screening of nemertea species for anabaseine activity [17]. Among the 14 species thatwere investigated, three stood out—*Lineus ruber, L. sanguineus*, and *L. viridis*, exhibiting crayfish paralytic activity that was far beyond what could be explained by anabaseine content. The *Lineus* toxin was described as a polypeptide neurotoxin, corresponding to Bacq’s “nemertine” [32,33]. Size exclusion experiments on these polypeptides indicated a molecular mass of approximately 3500 Da [5]. For *L. ruber* mucus extracts, it was shown that at least two separate polypeptide toxins were present. These were shown to cause spike activity in isolated lobster (*Homarus americanus*) preparations, whereas no effect was evident in a frog assay or in the hemolysis of human erythrocytes. The lack of material hampered further efforts into the nature and identity of these peptides.

**Figure 9:**
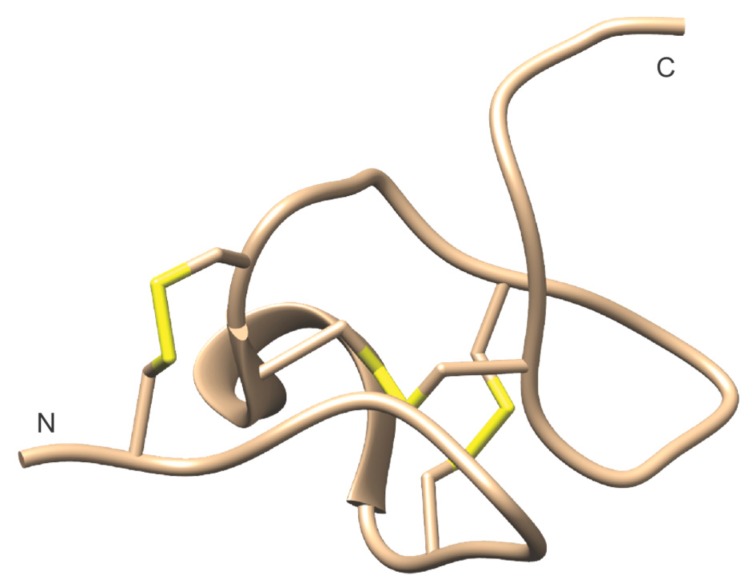
NMR structure of nemertide alpha-1 (RCSB id: 6ENA) [120].

More recently, *Lineus* nemerteans were explored as a production system for TTX [73]. No TTX could be identified, although the mucus was shown to be highly toxic to crustaceans [25]. Instead, the size exclusion of mucus from *L. longissimus* and subsequent UPLC-MS analysis led to the discovery of one peptide (named nemertide ß-1) closely resembling neurotoxin B-IV, and importantly, two peptides of lower mass (3308 and 3360, respectively) [120]. Although the relationship is not conclusive, these α-nemertides, as they were called, bear resemblance both in the size and toxicity to Bacq’s nemertine, as well as the *Lineus* peptides that were discovered by Kem. MALDI imaging, which was carried out on cross-sections of the nemertean, showed that the presence of all three peptides was focused to its epithelial/mucus layer. For further evaluation, initial focus was placed on the m/z 3308 peptide, designated nemertide α-1. The α-1 fraction was highly toxic when injected into *Carcinus maenas*, and the peptide was subsequently sequenced, synthesized, and its solution structure dwas etermined by 2D-NMR, Figure 9. α-1 has 31 residues containing two hydroxyprolines, and three disulfides forming an inhibitor cysteine knot motif in the peptide core. Sequence loops between cysteines are surface-exposed, and whereas the N-terminus is stabilized by a disulfide (Cys2–Cys16), the C-terminus appears to be flexible. The only segment of the regular secondary structure is a short α-helix in the loop between Cys-9 and Cys-15, and a series of tight turns. Three positions (Phe-8, Trp-22, Phe-24) make up a hydrophobic patch. The α-2 peptide differs only by a Val substitution of Phe-8.

α-1 was shown to be extremely potent. Injections into green crabs (*Carcinus maenas*) led to paralysis and death in sub nmol/kg doses, and comparable results were given by an *in vivo* assay on orange-spotted cockroach (*Blaptica dubia*). Voltage-clamp experiments using heterologously expressed voltage-gated sodium channels (VGSC) showed an EC_50_ value that was in the low nanomolar range for the German cockroach (*Blattella germanica*), and the activity was found to be selective for invertebrates (activity was also demonstrated on *Drosophila melanogaster* and *Varroa destructor*), as the corresponding experiment on mammalian VGSCs demonstrated an EC_50_ in the micromolar range, with certain subtype specificities. The *B. germanica* experiments also generated the hypothesis that α-1 acts via binding to the so-called site three, thereby inhibiting the inactivation of this channel [121], leading to constitutive activity. Some additional preliminary activity data [122], using an *Artemia salina* bioassay, [123] indicates that the nemertides α-2-6 exhibit similar toxicities to α-1 (EC_50_ ranges: 0.5–5 μM).

An overview of peptide toxins that were detected and characterized in nemerteans is displayed in Table 5.

#### 5.3.4. Other Peptide and Protein Toxins

The impact of molecular biology is only beginning to take its effect on the progress in nemertea toxin research. A milestone was the recent publication of the first full genome of a ribbon worm, the heteronemertean *Notospermus geniculatus* [15]. Prior to this, a limited number of papers have made use of genomic methods to discover new peptide toxins, notably in the aforementioned work by Butala et al. [119], leading to the discovery of additional parborlysin sequences. Whelan et al. analysed the transcriptomes from nine nemertean species (the heteronemerteans *Cerebratulus marginatus, Lineus lacteus, Lineus longissimus, Lineus ruber*, the hoplonemerteans *Malacobdella grossa, Paranemertes peregrine*, and the palaeonemerteans *Tubulanus polymorphus, Cephalothrix hongkongiensis*, and *Cephalothrix linearis*), finding an abundance of sequences that were identical or similar to those of known toxins [124]. Cytotoxin A-III was found in the four heteronemerteans, but the most prominent sequence was that of plancitoxin-1, which was present in all species. Plancitoxin-1, a DNase II hepatotoxin that is found in the crown-of-thorns starfish (*Acanthaster planci*) [125], was suggested to be secreted with the mucus to improve the effect of other toxin peptides, rather than acting on its own. Sequences that were related to the SNTX/VTX protein family (either of neoverrucotoxin, verrucotoxin, or stonustoxin), were also found in all of the sequences. These proteins have cytolytic properties, but their functional roles in nemerteans remain unclear. Interestingly, two Shk toxin-like sequences were found, a pseudechetoxin-like venom protein (*M. grossa*, *P. peregrina*) and that of a Cys-rich venom protein ENH1 (*C. linearis*). Shk toxins have been shown to act as potassium channel blockers, thus attracting interest as pharmaceutical targets, e.g., against auto-immune reactions [126]. SE-cephalotoxin genes were found in six species (all but *M. grossa, P. peregrine*, and *T. polymorphus*). SE-cephalotoxin appears to have a role in predation, having been found in the salivary glands of the cuttlefish (*Sepia esculenta*), although its biochemical role is unclear [127]. Similar distribution was found for sequences related to the haemolytic echotoxin-2. Two species (*C. hongkongiensis* and *C. marginatus*) exhibited natterin-4-related sequences, containing an aerolysin segment. This domain has been linked to edema and nociception [128]. In addition, two species (*C. hongkongiensis* and *C. linearis*) contained MACPF (membrane attack complex/perforin) toxin genes (PsTX 60B and ATX-60-A), which are known to have a variety of roles but most commonly as components in native immunity [129]. In the heteronemertean *Notospermus geniculatus* genome [15], 63 putative toxin genes were annotated. Fifteen of these were shared with other lophotrochozoans without reported toxicity and they are not further discussed here, but 26 sequences were found to be differentially expressed in eggs and tissues. In many cases, variants of toxin sequences that were found in Whelan’s study were also found in the *N. geniculatus* genome, such as cytotoxin A-III, plancitoxin-1, a number of SNTX/VTX protein or Shk-protein related sequences, and natterin sequences. Moreover, several neurotoxin-related sequences that were not previously found in nemerteans, such as delta-actitoxin-Amc1a, Mu-theraphotoxin-Hhn2a 4, perivitellin-2, and turripeptide Gsg9.2 were found, and a series of sequences involved in various aspects of blood coagulation. A handful of putative toxin sequences for which the function is presently poorly known was also found.

The proteins themselves were not identified in these studies, and little can be said regarding the relevance and actual roles of these proteins for nemerteans. However, it indicates that there is much yet to discover. This is further stressed by the aforementioned recent study by Jacobsson et al. [120], who performed analyses of 17 transcriptomes, some of which are previously reported [119,124,130]. This analysis revealed seven novel α-nemertides, eight novel β-nemertide sequences (neurotoxin B-analogs), and 29 parborlysin related sequences.

Although limitations do exist in terms of finding completely new toxin families while using data mining alone, the above examples demonstrate how molecular biology techniques without doubt will accelerate the toxin discovery rate within the nemertean phylum. Putative toxin gene sequences that are found in nemertean genomes and transcriptomes are summarized in Table 6.

## 6. The Ecological Roles of Nemertean Toxins

### 6.1. Nemerteans as Preys

On a large scale, nemerteans are rarely eaten by others. McDermott et al. [10] studied gut content in fish and birds, and generally found low percentages of nemerteans. Seemingly, some flat fish do eat nemerteans within *Cerebratulus* spp. Although some *Cerebratulus* species have been shown to have toxins, their effect on flat fish GI tract has not been studied, to the best of our knowledge. In addition, some species can be used as bait to catch fish, e.g., *Cerebratulus lacteus* [133], *Malacobdella* spp., and *Ototyphlonemertes brevis* [134], as well as *Polybrachiyorhynchus dayi* [135]. Conversely, other nemertean species (e.g., *Tubulanus annulatus*) are directly rejected should they be swallowed by fish (e.g., *Cottidae* spp., personal observation, Malin Strand). The puffer fish *Takifugu niphobles* has been shown to digest *Cephalothrix simula* [86]. It was suggested in this study that TTX from the nemertean might be accumulated in the fish (both are known to contain TTX). The puffer fish showed no appetite for *Baseodiscus curtus*, and in the same study, a goby fish rejected both nemertean species. A few echinoderms that digest some nemerteans seem ti ve unaffected; the small ophiuroid *Ophiocomina nigra* can swallow several specimens of *Lineus* spp. (viridis/ruber) and *Ramphogordius sanguineus* a day, and the same is true for the asteroid *Marthasterias glacialis* (personal observation, Malin Strand). Nemerteans also eat each other to some extent; for instance, *Lineus sanguineus* has been observed to ingest *Amphiporus lactifloreus* [136]. There is nothing in the literature that rejects the hypotheses that toxin production helps in avoiding becoming prey, although very few studies actually pinpoint specific responses between toxin production of a certain kind and predator defence. Moles et al. concluded that lipophilic extracts of the Antarctic species *Parborlasia corrugatus* displayed significant feeding deterrence [137] when presented to the common seastar *Odontaster validus*.

### 6.2. Nemerteans as Predators and Scavengers

Nemerteans are carnivorous and they feed on both dead and alive prey [134]. Some are scavenger species that eat through tissue suction, but current knowledge is that many nemerteans are voracious predators that actively hunt living preys [11]. The armed hoplonemerteans can locate and stab a prey with the stylet that is associated with toxins that act as both paralytic and tissue dissolving [138]. The prey is immobilized within seconds and the nemertean can then safely ingest the prey innards or the whole prey. Many hoplonemerteans seemingly prefer arthropods as prey, although there are observations on attacks of molluscs and other worm groups. The concentration of toxins to the proboscis of hoplonemerteans, as confirmed in the case of pyridine alkaloids [17], points to their role in predation.

Heteronemerteans, which lack the stylet, appear to be equally well-developed active predators. The proboscis is still useful to effectively immobilize or slow the locomotion of its polychaete prey, as demonstrated in the case of *Lineus sangineus* [139]. Brief contact between the prey and the everted proboscis is seemingly enough to affect the prey’s mobility. The proboscis of *L. sanguineus* is equipped with secretory cells with rod-shaped structures. These may serve to grip and possibly puncture prey, allowing for toxin entry (although the toxin used has not been characterized). Another example is *Cerebratulus lacteus*, which was studied in a soft-shell clam (*Mya arenaria*)-community where surface exploration was directly associated with the presence of prey [140]. The attack does not even necessarily involve proboscis evertion; the prey (in this case, a fairly slow moving bivalve) can simply be located and consumed by swallowing. Similarly, the predatory behaviour of *Riseriellus occultus* both in field observations and laboratory experiments was elegantly documented by Beckers et al. [141]. The nemertean was demonstrated to sneak into the soft tissue of a *Patella* gastropod and start eating without provoking any discernable counter reaction. In the case of *Gibbula umbilicalis*, the flat top shell, the attack was followed in close detail. The nemertean first attacked the snail foot, which then retracted into the mantle cavity, with the proboscis clinging onto it. The proboscis was thereby positioned next to the gills. Shortly thereafter, froth bubbles, which were interpreted as a protective reaction, emerged. A little while later, the foot retractor relaxed, suggesting a function of a toxin, and the nemertean moved in towards the apex of the shell, from where it digested the snail. 

A number of organisms have been found to co-exist in or on nemerteans in a symbiontic relationship, including *Haplosporidia* and *Microsporidia* protists and *Conoidasida* [142]. Whether these have a role for predation is unclear, however for TTX containing bacterial symbionts, this might be the case [62].

### 6.3. Commensal Nemerteans

Approximately 40 species, most of which hoplonemerteans within Malacobdellidae, Carcinonemertidae, Tetrastemmatidae, and Emplectonematidae, are considered to be commensal species [143], but whether the nemertean is mutualistic or parasitic is often unclear. Six species within Malacobdellidae have been found in the mantle cavities of bivalves, and studies on *Malacobdella grossa* [144] suggested that it does not harm its host, supporting the classification of Malacobdellidae as endocommensals. In contrast, some *Carcinonemertes* species are ectocommensal specialized egg predators of decapod crustaceans [145], using the stylet to pierce egg coats to access yolk. This is not exclusive for *Carcinonemertes*, and egg predation has been suggested to sometimes emerge as outbreaks of epidemic levels, causing heavy crustacean brood mortality [146].

The benefit of toxin production in commensal nemerteans is not obvious, but toxins can potentially provide the host with protection from other predators. No functional studies that are dedicated to toxins of commensal nemerteans appear to exist, but toxin presence has nevertheless been indicated in a few studies. The Whelan transcriptome study [124] demonstrated the presence of a number of putative toxin sequences in *Malacobdella grossa*, such as neoverrucotin subunit beta, a pseudechetoxin-like cysteine-rich venom protein, and plancitoxin-1. *M. grossa* has been the subject of study for toxin content in some other studies: the trancriptome analysis by Jacobsson et al. [120] did not indicated the presence of either alpha- or beta-nemertide and neither parborlysin sequences. Neither was Kem et al. able to find pyridine alkaloids in *M. grossa* [5,17], and TTX also appears to be absent [80]. Apparently, the most commonly known nemertean toxins are absent or present in very low amounts in these nemerteans. However, Asakawa et al. [69] indicated the possible presence of 4,9-anhydro-TTX in *Malacobdella japonica*. Moreover, Kem screened a number of nemertean species for pyridine alkaloids. *Carcinonemertes errans* was not found to contain pyridine alkaloids, whereas the presence of “unidentified” compounds of pyridine type was indicated in *Tetrastemma candidum* and *Tetrastemma reticulatum* [38]. This observation is perhaps significant in light of the observation that *Tetrastemma worki* contains anabaseine [5,17].

## 7. Conclusions and Future Outlook

The aim of this review was to provide a complete account of nemertean toxins that are known to date. Currently, three main groups of toxins can be distinguished: pyridine alkaloids, TTX and derivatives thereof, and peptides.

Pyridine alkaloids have mainly been found in hoplonemerteans, but the discovery efforts are scarce. The perhaps most extensive study on this topic was published in 1988 [38]. Around 15 related compounds were left uncharacterized in this study, suggesting that there is yet more to be found. Technical improvements over 30 years allow for the characterization of significantly smaller amounts, so time has come for further studies of nemertean pyridine alkaloids. In contrast, TTX and related compounds in nemerteans is an area of active research. Most likely, TTX is not produced by nemerteans themselves, and the role of the compound in nemerteans is unclear. Although most studies demonstrating the presence of TTX in nemerteans analyzed specimens from the Sea of Japan or Peter the Great Bay, it is noteworthy that TTX carrying nemerteans recently emerged in European waters [85]. Studies to understand the mechanisms of this “TTX migration” would be of great value. A large number of putative peptide and protein toxin genes have been accounted for in this review. However, at the peptide level, merely three main families of peptide toxins have been confirmed: neurotoxins of about 3 kDa; neurotoxins of about 6 kDa; and, cytolytic peptides in the size range 8–10 kDa. The exceptional toxicity of the neurotoxins suggests that they may find practical use, and we predict that this will undoubtedly attract more research in the area. As the arsenal of nemertean peptide toxins is growing, a comment on nomenclature is warranted. We have introduced the term nemertide [120] and propose that this could be employed as a general term for nemertean peptide toxins; Greek prefixes are then used to indicate families. Thus, the α-nemertides is used for the family of 3 kDa cystine knot neurotoxins, and we argue that the ß-nemertides (6 kDa), in effect, can be viewed as a part of the same family as the neurotoxins BI to BIV. Similarly, the parborlysins likely belong to the same family as cytotoxins AI to AIV.

The development of marine natural products chemistry can be illustrated by the number of compounds isolated/discovered each year (one in 1963 vs. 1277 in 2016) [147,148], with the last 15 years seeing an average increase of approximately 50 compounds per annum. From this body of work, seven out of 1453 FDA-approved drugs as of 2013 were of marine origin [149]. Current progress is substantial, and today an abundance of pharmacologically active compounds are being evaluated for potential medicinal use [150,151,152].

**Figure 10:**
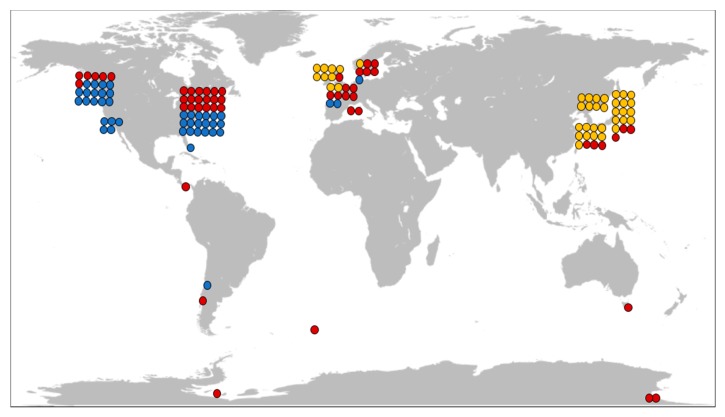
Schematic illustration of sampling sites for nemerteans analyzed with regard to toxin content reported in scientific publications. Each dot represents any unique species investigated in one report (if the same sample was interpreted as being used in several studies, it is still one dot). Blue dots correspond to pyridine alkaloids, yellow dots: TTX, and red dots: peptide toxins.

Only a minute number of those natural products originate from the nemertea phylum. However, as shown in this review, it is clear that nemerteans represent a promising source of bioactive compounds, given that less than 5% of the world’s ribbon worm species have been analyzed for toxin content to any extent. Moreover, in no single case can it be stated that a complete analysis, which takes into account both macromolecular toxins and small molecules, has been carried out. There is also a geographical limitation to the efforts. Sampling has been focused to a limited number of sites and, at this point, it appears that no nemertean species have been sampled in Africa nor India, and only one from Oceania and a few examples from South America have ever been investigated in this context, Figure 10. Only one terrestrial species has been investigated, and no fresh water species. We are only beginning to uncover the chemistry of nemertean worms.

Hence, what lies in the future of nemertean toxins? Whereas TTX has been an important tool in the characterization of sodium channels and offers promise as a drug lead [153], it is doubtful that nemerteans will be a source for large-scale production of TTX, unless of course enzymes that are involved in its biosynthesis could be identified. In the case of pyridine alkaloids, DMXBA has been extensively investigated, and its medicinal potential is still under investigation [52]. As for peptide toxins, the use of the α-nemertides in pesticide applications indeed appears to be feasible [122]. It is difficult to predict the outcome of nemertean toxins, but it is clear that they provide an intriguing addition to the field of toxin research. Taking into consideration that the present status has been achieved through a research volume of little more than 100 published scientific articles, ample opportunities for discovery should lie ahead.

## Figures and Tables

**Table 1:** Biological activities of nemertean pyridyl alkaloids (data from [41]).

Compound	Barnacle Larvae Settlement Inhibition IC_50_ (μM)	Barnacle Larvae Median Lethal Concentration IL_50_ (μM)	Crayfish Acute Paralytic Dose PD_50_ (μg)
2,3′-bipyridyl	4.1 (3.2–5.3) ^a^	1.9 (1.0–4.3)	0.88 (0.71–1.1)
Anabaseine	1.2 (0.91–1.7)	2	3.6 (3.1–4.1)
Nemertelline	3.2 (1.8–6.0)	-	>120
Anabasine	3.0 (1.5–4.9)	-	3.9 (3.4–4.5)

^a^ Parentheses indicate standard deviations.

**Table 2:** Overview of nemertean species analyzed for anabaseine related compounds.

Species	Origin	Toxin	Sample	Extraction and Analysis	Source
Class Hoplonemertea	Order Monostilifera				
*Amphiporus angulatus*	NH + ME shores, USA	Anabaseine	Whole body	TLC - alkal CHCl_3_ extr., DMAB deriv.	[5,17]
*Amphiporus angulatus*	NH + ME shores, USA	Anabaseine	Whole body	Chromatogr - alkal CHCl_3_ extr	[36]
*Amphiporus angulatus*	NH + ME shores, USA	Nemertelline	Whole body	Chromatogr - alkal CHCl_3_ extr	[36]
*Amphiporus angulatus*	NH + ME shores, USA	2,3′-bipyridyl	Whole body	Chromatogr - alkal CHCl_3_ extr	[36]
*Amphiporus angulatus*	NH + ME shores, USA	3-methyl-2,3′-bipyridyl	Whole body	Chromatogr - alkal CHCl_3_ extr	[36,40]
*Amphiporus angulatus*	Not stated	Bipyridyl toxins	Live animal	Not stated	[53]
*Amphiporus angulatus*	Eastport, MA, USA.	2,3′-bipyridyl, nemertelline + 4 unidentified	Whole body Frozen	TLC - alkal/acid CHCl_3_ extr	[38]
*Amphiporus bimaculatus*	San Juan Island, Washington, USA.	Unidentified pyridyl	Whole body Frozen	TLC - alkal/acid CHCl_3_ extr	[38]
*Amphiporus cruentatus*	Woods hole, MA, USA.	5 unidentified	Whole body Frozen	TLC - alkal/acid CHCl_3_ extr	[38]
*Amphiporus formidabilis*	San Juan Island, Washington, USA.	3 unidentified	Whole body Frozen	TLC - alkal/acid CHCl_3_ extr	[38]
*Amphiporus lactifloreus*	Not stated	Low anabaseine activity	Whole body	TLC - alkal CHCl_3_ extr., DMAB deriv.	[17]
*Amphiporus lactifloreus*	Not stated	Low anabaseine activity	Whole body	TLC - alkal CHCl_3_ extr., DMAB deriv.	[5]
*Amphiporus lactifloreus*	Bangor, Wales, UK	Anabasine	Whole body Frozen	TLC - alkal/acid CHCl_3_ extr	[38]
*Amphiporus ochraceus*	Not stated	None found	Whole body	TLC - alkal CHCl_3_ extr., DMAB deriv.	[5,17]
*Amphiporus ochraceus*	Woods hole, MA, USA	5 unidentified	Whole body Frozen	TLC - alkal/acid CHCl_3_ extr	[38]
*Argonemertes dendyi*	Tomales bay, CA, USA	1 unidentified	Whole body Frozen	TLC - alkal/acid CHCl_3_ extr	[38]
*Carcinonemertes errans*	Bodega bay, CA, USA	None found	Whole body Frozen	TLC - alkal/acid CHCl_3_ extr	[38]
*Emplectonema gracile*	San Juan Island, WA, USA	Possibly 1 unidentified	Whole body Frozen	TLC - alkal/acid CHCl_3_ extr	[38]
*Emplectonema gracile*	San Juan Island, WA, USA	1 unidentified	Whole body Frozen	TLC - alkal/acid CHCl_3_ extr	[38]
*Emplectonema neesi*	Bangor, Wales, UK	None found	Whole body Frozen	TLC - alkal/acid CHCl_3_ extr	[38]
*Fasciculonemertes arenicola*	Los Molles, Chile	5 unidentified	Whole body Frozen	TLC - alkal/acid CHCl_3_ extr	[38]
*Geonemertes pelaensis*	Miami, FL, USA	1 unidentified	Whole body Frozen	TLC - alkal/acid CHCl_3_ extr	[38]
*Malacobdella grossa*	Not stated	None found	Whole body	TLC - alkal CHCl_3_ extr - DMAB deriv	[5,17]
*Nipponnemertes pulchra ^a^*	Helsingør, Dk	1 unidentified	Whole body Frozen	TLC - alkal/acid CHCl_3_ extr	[38]
*Paranemertes peregrina*	San Juan Island, WA, USA	Anabaseine	Whole body	Al_2_O_3_ chrom-alkal CHCl_3_ extr.	[35]
*Paranemertes peregrina*	San Juan Island, WA, USA	Anabaseine	Body w.o. proboscis	MS + UV - DMAB deriv	[5,17]
*Paranemertes peregrina*	San Juan Island, WA, USA	Anabaseine	Anterior proboscis	MS + UV - DMAB deriv	[5,17]
*Paranemertes peregrina*	San Juan Island, WA, USA	Anabaseine	Median proboscis	MS + UV - DMAB deriv	[5,17]
*Paranemertes peregrina*	San Juan Island, WA, USA	Anabaseine	Posterior proboscis	MS + UV - DMAB deriv	[5,17]
*Paranemertes peregrina*	San Juan Island, WA, USA	Anabaseine	Periph part body wall	MS + UV - DMAB deriv	[17]
*Paranemertes peregrina*	San Juan Island, WA, USA	Anabaseine	Periph part body wall	MS + UV - DMAB deriv	[5]
*Paranemertes peregrina*	San Juan Island, WA, USA	Low anabaseine	Body core tissues	MS + UV - DMAB deriv	[17]
*Paranemertes peregrina*	San Juan Island, WA, USA	Low anabaseine	Body core tissues	MS + UV - DMAB deriv	[5]
*Paranemertes peregrina*	San Juan Island, WA, USA	Anabaseine	Whole body	TLC - alkal CHCl_3_ extr - DMAB deriv	[5,17]
*Paranemertes peregrina*	Bodega Bay, CA, USA	2 unidentified	Whole body Frozen	TLC - alkal/acid CHCl_3_ extr	[38]
*Prosadenoporus californiensis ^b^*	Tomales Bay, CA, USA	2 unidentified	Whole body Frozen	TLC - alkal/acid CHCl_3_ extr	[38]
*Prostoma graecense ^c^*	Not stated	Possibly anabaseine	Whole body	TLC - alkal CHCl_3_ extr - DMAB deriv	[5,17]
*Tetrastemma candidum*	Woods hole, CA, USA	1 unidentified	Whole body Frozen	TLC - alkal/acid CHCl_3_ extr	[38]
*Tetrastemma reticulatum*	San Juan Island, WA, USA	1 unidentified	Whole body Frozen	TLC - alkal/acid CHCl_3_ extr	[38]
*Tetrastemma worki*	Not stated	Anabaseine	Whole body	TLC - alkal CHCl_3_ extr - DMAB deriv	[5,17]
*Zygonemertes virescens*	Not stated	None found	Whole body	TLC - alkal CHCl_3_ extr - DMAB deriv	[5]
*Zygonemertes virescens*	Woods hole, MA, USA	Anabasine	Whole body Frozen	TLC - alkal/acid CHCl_3_ extr	[38]
Class Palaeonemertea					
*Carinoma* sp.	Not stated	None found	Whole body	TLC - alkal CHCl_3_ extr., DMAB deriv.	[5]
*Carinoma tremaphoros ^d^*	Woods hole, MA, USA.	Not reported	Whole body Frozen	TLC - alkal/acid CHCl_3_ extr	[38]
*Cephalothrix spiralis ^d,e^*	Not stated	None found	Whole body	TLC - alkal CHCl_3_ extr., DMAB deriv.	[5,17]
Class Pilidiophora	Order Heteronemertea				
*Cerebratulus lacteus*	Woods hole, MA, USA.	None found	Whole body	TLC - alkal CHCl_3_ extr., DMAB deriv.	[5,17]
*Cerebratulus lacteus*	Boston, MA, USA	None found	Whole body Frozen	TLC - alkal/acid CHCl_3_ extr	[38]
*Lineus ruber*	Not stated	Anabaseine/Nemertine Below detection level	Whole body	TLC - alkal CHCl_3_ extr., DMAB deriv.	[17]
*Lineus ruber*	NH + ME shores, USA	Anabaseine/Nemertine Below detection level	Whole body	TLC - alkal CHCl_3_ extr., DMAB deriv.	[5]
*Lineus sanguineus ^f^*	Not stated	Anabaseine/Nemertine Below detection level	Whole body	TLC - alkal CHCl_3_ extr., DMAB deriv.	[5,17]
*Lineus viridis*	Not stated	Anabaseine/Nemertine	Whole body	TLC - alkal CHCl_3_ extr., DMAB deriv.	[17]
*Lineus viridis*	NH + ME shores, USA	Anabaseine/Nemertine	Whole body	TLC - alkal CHCl_3_ extr., DMAB deriv.	[5]
*Lineus viridis*	Woods hole, MA, USA	None found	Whole body Frozen	TLC - alkal/acid CHCl_3_ extr	[38]
*Micrura leidyi*	Not stated	Anabaseine/Nemertine	Whole body	TLC - alkal CHCl_3_ extr - DMAB deriv	[17]
*Micrura leidyi*	Woods hole, MA, USA	None found	Whole body Frozen	TLC - alkal CHCl_3_ extr	[38]
*Micrura leidyi*	Not stated	Anabaseine/Nemertine Below detection level	Whole body	TLC - alkal CHCl_3_ extr - DMAB deriv	[5]
*Parvicirrus dubius ^g^*	Not stated	Anabaseine/Nemertine Below detection level	Whole body	TLC - alkal CHCl_3_ extr - DMAB deriv	[5]
*Siphonenteron bicolour ^h^*	Not stated	Anabaseine/Nemertine Below detection level	Whole body	TLC - alkal CHCl_3_ extr - DMAB deriv	[5]
*Siphonenteron bicolour ^h^*	Woods hole, MA, USA	None found	Whole body Frozen	TLC - alkal/acid CHCl_3_ extr	[38]

Abbreviations: Alkal—alkaline; Chrom—chromatography; DMAB deriv—p-dimethyl aminobenzylidene deriative; Extr—extraction; MS—mass spectrometry; TLC—thin layer chromatography; UV—ultraviolet spectroscopy. ^a^ In source referred to as *Nipponemertes pulcher* (synonymous). ^b^ In source referred to as *Pantinonemertes californiensis* (synonymous). ^c^ In source referred to as *Prostoma rubrum* (synonymous). ^d^ Used as negative control. ^e^ In source referred to as *Procephalothrix spiralis* (synonymous). ^f^ In source referred to as *Lineus socialis* (synonymous). ^g^ In source referred to as *Lineus dubius* (synonymous). ^h^ In source referred to as *Lineus bicolor* (synonymous). Species are denoted according to WoRMS, World Register of Marine Species as of 19-02-11 [54].

**Table 3:** Nemertean species analyzed for TTX content.

Species	Origin	Toxin/-s	Sample	Extraction and Analysis	Ref
Class Hoplonemertea	Order Monostilifera				
*Amphiporus lactifloreus*	Llandudno, Wales, UK, or Rhosneigr, Wales, UK	TTX-like cpds	Acidic whole body extract	UV spectroscopy and HPLC	[72]
*Amphiporus* sp.	Akkeshi Bay, Hokkaido, Jpn	TTX + analogs	Acidic whole body extract	Defatted, charcoal purif, HPLC and GC-MS-C9 base	[69]
*Nipponnemertes bimaculata ^a^*	Peter the Great Bay, Rus/Jpn	Very low TTX	Acidic MeOH extract	HPLC-MS/MS	[83]
*Malacobdella japonica*	Akkeshi Bay, Hokkaido, Jpn	TTX + analogs Anhydro-TTX	Acidic whole body extract	Defatted, charcoal purif, HPLC, GC-MS-C9 base	[69]
*Malacobdella grossa*	Peter the Great Bay, Rus/Jpn	Antimicrobial activity. No TTX.	Bacteria - whole body homogenate	Confocal laser microscopy after TTX antibody labeling	[80]
*Nemertellina yamaokai*	Akkeshi Bay, Hokkaido, Jpn	None found	Acidic whole body extract	Defatted, charcoal purif, HPLC, GC-MS-C9 base	[69]
*Nipponnemertes punctatula*	Otsuchi Bay, Iwate, Jpn	TTX, epi-, anhydro-TTX	Acidic whole body extract	Defatted, charcoal purif, HPLC, GC-MS-C9 base	[69]
*Paranemertes* sp.	Peter the Great Bay, Rus/Jpn	None found	Acidic methanol extract	HPLC-MS/MS	[83]
*Quasitetrastemma nigrifrons ^b^*	Akkeshi Bay, Hokkaido, Jpn	None found	Acidic whole body extract	Defatted, charcoal purif, HPLC, GC-MS-C9 base	[69]
*Quasitetrastemma stimpsoni ^c^*	Akkeshi Bay, Hokkaido, Jpn	(TTX and analogues) Not analyzed	Acidic whole body extract	Defatted, charcoal purif, HPLC, GC-MS-C9 base	[69]
*Quasitetrastemma stimpsoni ^c^*	Peter the Great Bay, Rus/Jpn	Bact. cultures Antimicrob activity. TTX.	Bacteria from whole body homogenate	Confocal laser microscopy after TTX antibody labeling	[80]
*Quasitetrastemma stimpsoni*	Peter the Great Bay, Rus/Jpn	Very low TTX	Acidic methanol extract	HPLC-MS/MS	[83]
Class Palaeonemertea					
*Cephalothrix linearis*	Shimoda, Shizuoka, Jpn	TDA-like, TTX, anhydro-, epi-TTX	Proboscis, body	SEC, TLC, el.phoresis, HPLC, GC-C9 base	[65]
*Cephalothrix linearis*	Shimoda, Shizuoka, Jpn	TDA-like	Handling stimulus secretion	SEC, TLC, el.phoresis, HPLC, GC-C9 base	[65,66]
*Cephalothrix rufifrons*	Llandudno, Wales, UK, or Rhosneigr, Wales, UK	TTX-like cpds	Acidic whole body extract	UV spectroscopy and HPLC	[72]
*Cephalotrix rufifrons ^d^*	Cornwall, UK	None in extract, but TTX in bacteria isolate	Acidic whole body extract and bacteria isolates	HPLC-MS/MS of extract and isolates	[85]
*Cephalothrix simula*	Hiroshima Bay, Jpn	TTX, epi-, anhydro-TTX	Acidic whole body extract	Defatted, charcoal purif, HPLC and GC-MS-C9 base	[69]
*Cephalothrix simula*	Akkeshi Bay, Hokkaido, Jpn	TTX, epi-, anhydro-TTX	Acidic whole body extract	Defatted, charcoal purif, HPLC and GC-MS-C9 base	[69]
*Cephalothrix simula*	Otsuchi Bay, Iwate, Jpn	TTX, epi-, anhydro-TTX	Acidic whole body extract	Defatted, charcoal purif, HPLC and GC-MS- C9 base	[69]
*Cephalothrix simula ^e^*	Peter the Great Bay, Rus/Jpn	TTX-*Bacillus* sp.	Bacteria isolates	Immunovisualization	[78,79]
*Cephalothrix simula*	Peter the Great Bay, Rus/Jpn	7 TTX derivatives	Acidic MeOH extract	HPLC-MS/MS	[83]
*Cephalotrix simula*	Cornwall, UK	TTX and derivatives	Acidic whole body extract and bacteria isolates	HPLC-MS/MS of extract and isolates	[85]
*Cephalothrix* sp.	Hiroshima Bay, Jpn	TTX, epi-, anhydro-TTX	Acidic whole body extract	Defatted, SEC, IEC. TLC, HPLC, GCMS-C9 base	[67]
*Cephalothrix* sp.	Hiroshima Bay, Jpn	TTX, epi-, anhydro-TTX	Whole body, frozen	Activated charcoal, SEC, IEC, cryst from acidic CH_3_OH soln, GCMS-C9 base, NMR + MS	[68]
*Cephalothrix* sp.	Hiroshima Bay, Jpn	TTX	Whole body, cross-section	Anti-TTX antibodies	[81]
*Tubulanus annulatus*	Cornwall, UK	None found	Acidic whole body extract and bacteria isolates	HPLC-MS/MS of extract and isolates	[85]
*Tubulanus polymorphus ^f^*	Not stated	TTX	Not stated	Immunostaining	[87]
*Tubulanus punctatus*	Seto Inland sea Hiroshima, Jpn	Anhydro-TTX	Whole body	Defatted, charcoal purif, HPLC, GC-MS-C9 base	[64]
*Tubulanus punctatus*	Peter the Great Bay, Rus/Jpn	Very low TTX	Acidic MeOH extract	HPLC-MS/MS	[83]
Class Pilidiophora	Order Heteronemertea				
*Cerebratulus marginatus*	Peter the Great Bay, Rus/Jpn	None found	Acidic MeOH extract	HPLC-MS/MS	[83]
*Dushia atra ^f^*	Not stated	TTX	Not stated	Immunostaining	[87,88]
*Kulikovia alborostrata*	Peter the Great Bay, Rus/Jpn	Very low TTX	Acidic MeOH extract	HPLC-MS/MS	[83]
*Kulikovia manchenkoi*	Peter the Great Bay, Rus/Jpn	TTX + 3 deriv	Acidic MeOH extract	HPLC-MS/MS	[83]
*Lineus alborostratus*	Akkeshi Bay, Hokkaido, Jpn	TTX, anhydro-, epi-TTX	Acidic whole body extract	Defatted, charcoal purif, HPLC, GC-MS-C9 base	[69]
*Lineus alborostratus*	Peter the Great Bay, Rus/Jpn	TTX	Whole body	Immunostaining	[82]
*Lineus alborostratus*	Peter the Great Bay, Rus/Jpn	Bact cultured for TTX. Antimicrob activity.	Bacteria from whole body homogenate	Confocal laser microscopy after TTX antibody labeling	[80]
*Lineus bilineatus*	Akkeshi Bay, Hokkaido, Jpn	None found	Acidic whole body extract	Defatted, charcoal purif, HPLC, GC-MS-C9 base	[69]
*Lineus fuscoviridis*	Seto Inland sea, Hiroshima, Jpn	TTX, anhydro-TTX	Whole body	Defatted, SEC, IEC. TLC, HPLC., GC-MS-C9 base	[64]
*Lineus longissimus*	Llandudno, Wales, UK, or Rhosneigr, Wales, UK	TTX-like cpds	Acidic whole body extract and mucus	UV spectroscopy and HPLC	[72]
*Lineus longissimus*	Koster Fiord, Swe, and Millport, Scot, UK	<5 kDa cpd	Mucus + *Vibrio* cultures	Various purification methods	[25]
*Lineus ruber*	Llandudno, Wales, UK, or Rhosneigr, Wales, UK	TTX-like cpds	Acidic whole body extract	UV spectroscopy and HPLC	[72]
*Lineus sanguineus ^g^*	Llandudno, Wales, UK, or Rhosneigr, Wales, UK	TTX-like cpds	Acidic whole body extract	UV spectroscopy and HPLC	[72]
*Lineus torquatus*	Akkeshi Bay, Hokkaido, Jpn	TTX, anhydro-, epi-TTX	Acidic whole body extract	Defatted, charcoal purif, HPLC, GC-MS-C9 base	[69]
*Lineus viridis*	Llandudno, Wales, UK, or Rhosneigr, Wales, UK	TTX-like cpds	Acidic whole body extract	UV spectroscopy and HPLC	[72]
*Micrura akkeshiensis*	Akkeshi Bay, Hokkaido, Jpn	None found	Acidic whole body extract	Defatted, charcoal purif, HPLC, GC-MS-C9 base	[69]
*Micrura bella*	Peter the Great Bay, Rus/Jpn	None found	Acidic MeOH extract	HPLC-MS/MS	[83]
*Micrura verrilli ^f^*	Not stated	TTX	Not stated	Immunostaining	[87,88]
*Nipponomicrura uchidai*	Peter the Great Bay, Rus/Jpn	None found	Acidic methanol extract	HPLC-MS/MS	[83]
*Riseriellus occultus*	Llandudno, Wales, UK, or Rhosneigr, Wales, UK	TTX-like cpds	Acidic whole body extract	UV spectroscopy and HPLC	[72]
*Yininemertes pratensis*	Haengjunaru, Han river Estuary, South Korea	TTX + analogs, derivatives. Tox cpd mass 291.1	EtOH extract	Dry homogenate lysis in pure EtOH, hydrophobic HPLC, MALDI-TOF	[84]
Class Pilidiophora	Genus Hubrechtella				
*Hubrechtella juliae*	Peter the Great Bay, Rus/Jpn	Bact cultured for TTX. Antimicrob activity.	Bacteria from whole body homogenate	Confocal laser microscopy after TTX antibody labeling	[80]

Abbreviations: Cpd—compound; Cryst—crystallization; Deriv—derivatization/derivative; El.phoresis—electrophoresis; EtOH; ethanol; GC-MS-C9 base—gas chromatography—mass spectrometry of TTX C9 base derivative; IEC—ion-exchange chromatography; MeOH—methanol; Purif —purification; SEC—size exclusion chromatography (gel filtration); TDA—tetrodonic acid. ^a^ In source referred to as *Collarenemertes bimaculata.* ^b^ In source referred to as *Tetrastemma nigrifrons* (synonymous). ^c^ In source referred to as *Tetrastemma stimpsoni* (synonymous). ^d^ In source referred to as *Cephalothrix rubifrons.* ^e^ Host organism for bacteria claimed to contain TTX. ^f^ Conference abstract only. ^g^ In source referred to as *Ramphogordius sanguineus* (synonymous). Species are denoted according to WoRMS, World Register of Marine Species as of 19-02-11 [54].

**Table 4:** Summary of neurotoxin B-IV mutant analyses.

Residue	AA	Modification	Effect	Source
1	Ala	Extra Met-1	35-40% of native	[100]
3	Ala	Ser (+8 Ser)	x2 activity	[101]
		Gly (+8 Gly)	Slightly less active	[101]
5	Trp	HNB ^a^ w Trp 30, 1 eq.	Slightly less active	[92]
		HNB w Trp 30, 2 eq.	Inactive	[93]
8	Ala	Ser (+8 Ser)	x2 activity	[101]
		Gly (+8 Gly)	Slightly less active	[101]
9	Tyr	Nitration	Inactive	[92]
10	Hyp	Pro	Active	[101]
12	Cys	Reduction (all Cys)	Inactive	[94]
13	Glu	Gly	Active	[102]
		Ala	Active	[102]
		Gln	Active	[102]
16	Cys	Reduction (all Cys)	Inactive	[94]
17	Arg	Gln	Inactive	[102]
		Ala	Inactive	[102]
		Lys	Inactive	[102]
18	Lys	Gln	Active	[103]
		Gln + Gln 19	Slightly less active	[103]
19	Lys	Gln + Gln 18	Slightly less active	[103]
21	Asp	Ala	Active	[102]
		Asn	Active	[102]
		Pro	75% helix reduction. 10-fold reduction in activity	[102]
22	Leu	Asp	Active	[103]
23	Cys	Reduction (all Cys)	Inactive	[94]
25	Arg	Gln	400-fold reduction	[102]
		Lys	Slightly less active	[102]
26	Cys	Reduction (all Cys)	Inactive	[94]
29	Lys	Asn	Active	[103]
30	Trp	HNB	Inactive	[93]
		Ser	40-fold reduction	[103]
		Tyr	Active	[103]
		Phe	Active	[103]
		HNB with Trp 5, 1 eq.	Slightly less active	[93]
		HNB with Trp 5, 2 eq.	Inactive	[93]
33	Lys	Asn	Active	[103]
34	Arg	Gln	20-fold reduction. Structure destabilized.	[102]
		Ala	80-fold reduction	[102]
37	Cys	Reduction (all Cys)	Inactive	[94]
41	Cys	Reduction (all Cys)	Inactive	[94]
48	Cys	Reduction (all Cys)	Inactive	[94]
52	Cys	Reduction (all Cys)	Inactive	[94]
53	Lys	Truncation	Active	[102]
54	Lys	Truncation	Active	[102]
55	Glu	Truncation	Active	[102]

^a^ HNB: 2-hydroxy-5-nitrobenzyl bromide.

**Table 5:** Overview of peptide toxins found in nemerteans.

ID	Toxin	Species	Geographic Origin	Function	Ref
*Pore-forming/hemolytic*					
-	Cytotoxin A-I ^a^	*Cerebratulus lacteus*	Woods hole, MA, USA	Pore-forming; hemolytic	[5,104]
-	Cytotoxin A-II ^a^	*Cerebratulus lacteus*	Woods hole, MA, USA	Pore-forming; hemolytic	[5,104]
P01527	Cytotoxin A-III ^a^	*Cerebratulus lacteus*	Woods hole, MA, USA	Pore-forming; hemolytic	[5,104]
-	Cytotoxin A-IV ^a^	*Cerebratulus lacteus*	Woods hole, MA, USA	Pore-forming; hemolytic	[104]
6ENA ^b^	Nemertide α-1	*Lineus longissimus*	Koster Fiord, Swe	VGSC activator, paralytic	[120]
-	Nemertide α-2	*Lineus longissimus*	Koster Fiord, Swe	Likely VGSC activator, paralytic	[120]
-	Nemertide β-1	*Lineus longissimus*	Koster Fiord, Swe	Neurotoxin B-IV homolog. Likely paralytic.	[120]
-	”Nemertine”	*Lineus ruber*		Possible nemertide	[5]
-	”Nemertine”	*Tenuilineus bicolor ^f^*	Not stated	Possible nemertide	[5]
-	”Nemertine”	*Lineus viridis*	Not stated	Possible nemertide	[5]
-	<5 kDa component	*Lineus longissimus*	Koster Fiord, Swe, and Millport Scot, UK	Paralytic. Likely α-nemertide	[25]
-	Neurotoxin B-I ^c^	*Cerebratulus lacteus*	Long Island Sound, NY, USA	Likely paralytic	[89]
P01526	Neurotoxin B-II ^c^	*Cerebratulus lacteus*	Long Island Sound, NY, USA	Paralytic	[89]
P01526	Neurotoxin B-II	*Cerebratulus lacteus*	Woods hole, MA, USA	Paralytic	[104]
-	Neurotoxin B-III ^c^	*Cerebratulus lacteus*	Long Island Sound, NY, USA	Likely paralytic	[89]
P01525	Neurotoxin B-IV	*Cerebratulus lacteus*	Woods hole, MA, USA	Paralytic	[104]

ID is short for UniProtKB entry (uniprot.org). ^a^ In source referred to as Cerebratulus toxin A (I-IV). ^b^ PDB code. ^c^ In source referred to as Cerebratulus toxin B (I-IV). Species are denoted according to WoRMS, World Register of Marine Species as of 19-02-11 [54].

**Table 6:** Overview of putative toxin gene sequences found in nemerteans.

ID	Toxin Gene Homolog	Species	Geographic Origin	Proposed Function	Ref
*Pore-forming/hemolytic*					
P01527	Cytotoxin A-III	*Cerebratulus marginatus*	San Juan Island, WA, USA [130]	Pore-forming; hemolytic	[124]
P01527	Cytotoxin A-III	*Lineus lacteus ^a^*	Banyuls, Fréjus, Fra [131]	Pore-forming; hemolytic	[124]
P01527	Cytotoxin A-III	*Lineus longissimus*	Erdeven + Roscoff, Fra [131]	Pore-forming; hemolytic	[124]
P01527	Cytotoxin A-III	*Lineus ruber*	Roscoff, Wimereux, Fra [131]	Pore-forming; hemolytic	[124]
P01527	Cytotoxin A-III	*Notospermus geniculatus*	Ushimado Mar Inst Okayama Univ, Jpn	Pore-forming; hemolytic	[15]
Q54316	Hemolysin B	*Notospermus geniculatus*	Ushimado Mar Inst Okayama Univ, Jpn	Hemolytic	[15]
P54176	Hemolysin-3	*Notospermus geniculatus*	Ushimado Mar Inst Okayama Univ, Jpn	Hemolytic	[15]
A0A0N7HUN6	Parborlysin-4	*Parborlasia corrugatus*	Adelaide Island, Antarctica	Likely pore-forming, hemolytic	[119]
A0A0P0CC97	Parborlysin-5	*Parborlasia corrugatus*	Adelaide Island, Antarctica	Likely pore-forming, hemolytic	[119]
A0A0P0BUQ6	Parborlysin-6	*Parborlasia corrugatus*	Adelaide Island, Antarctica	Liekely pore-forming, hemolytic	[119]
A0A0P0CHY3	Parborlysin-7	*Parborlasia corrugatus*	Adelaide Island, Antarctica	Likely pore-forming, hemolytic	[119]
-	Parborlysin/cytotoxin homolog 1 (Locus_9778)	*Cerebratulus marginatus*	San Juan Island, WA, USA [124,130,132]	Likely pore-forming	[120]
-	Parborlysin/cytotoxin homolog 2 ( Locus_9778)	*Cerebratulus marginatus*	San Juan Island, WA, USA [124,130,132]	Likely pore-forming	[120]
-	Parborlysin/cytotoxin homolog 3 (Locus_40830)	*Hubrechtella ijimai*	Hamanko, Honshu, Jpn [132]	Likely pore-forming	[120]
-	Parborlysin/cytotoxin homolog 4 (comp17199)	*Lineus lacteus ^a^*	Banyuls, Fréjus, Fra [131]	Likely pore-forming	[120]
-	Parborlysin/cytotoxin homolog 5 (comp55821)	*Lineus lacteus ^a^*	Banyuls, Fréjus, Fra [131]	Likely pore-forming	[120]
-	Parborlysin/cytotoxin homolog 6 (Contig1463)	*Lineus lacteus ^a^*	Banyuls, Fréjus, Fra [131]	Likely pore-forming	[120]
-	Parborlysin/cytotoxin homolog 7 (Comp16298)	*Lineus lacteus ^a^*	Banyuls, Fréjus, Fra [131]	Likely pore-forming	[120]
-	Parborlysin/cytotoxin homolog 8 (Comp9226)	*Lineus lacteus ^a^*	Banyuls, Fréjus, Fra [131]	Likely pore-forming	[120]
-	Parborlysin/cytotoxin homolog 9 (contig46055)	*Lineus lacteus ^a^*	Banyuls, Fréjus, Fra [131]	Likely pore-forming	[120]
-	Parborlysin/cytotoxin homolog 10 (Comp45258)	*Lineus longissimus*	Koster Fiord, Swe	Likely pore-forming	[120]
-	Parborlysin/cytotoxin homolog 11 (comp48)	*Lineus longissimus*	Koster Fiord, Swe	Likely pore-forming	[120]
-	Parborlysin/cytotoxin homolog 12 (comp21702)	*Lineus longissimus*	Koster Fiord, Swe	Likely pore-forming	[120]
-	Parborlysin/cytotoxin homolog 13 (contig49129)	*Lineus longissimus*	Koster Fiord, Swe	Likely pore-forming	[120]
-	Parborlysin/cytotoxin homolog 14 (comp17823/17-134)	*Lineus longissimus*	Koster Fiord, Swe	Likely pore-forming	[120]
-	Parborlysin/cytotoxin homolog 15 (contig31748)	*Lineus longissimus*	Koster Fiord, Swe	Likely pore-forming	[120]
-	Parborlysin/cytotoxin homolog 16 (comp17823/17-147)	*Lineus longissimus*	Koster Fiord, Swe	Likely pore-forming	[120]
-	Parborlysin/cytotoxin homolog 17 (comp17823/17-145)	*Lineus longissimus*	Koster Fiord, Swe	Likely pore-forming	[120]
-	Parborlysin/cytotoxin homolog 17 (contig56815)	*Lineus longissimus*	Koster Fiord, Swe	Likely pore-forming	[120]
-	Parborlysin/cytotoxin homolog 18 (comp52392)	*Lineus ruber*	Roscoff, Wimereux, Fra [131]	Likely pore-forming	[120]
-	Parborlysin/cytotoxin homolog 19 (contig63996)	*Lineus ruber*	Roscoff, Wimereux, Fra [131]	Likely pore-forming	[120]
-	Parborlysin/cytotoxin homolog 20 (contig3234)	*Lineus ruber*	Roscoff, Wimereux, Fra [131]	Likely pore-forming	[120]
-	Parborlysin/cytotoxin homolog 21 (comp40150/seq2)	*Lineus ruber*	Roscoff, Wimereux, Fra [131]	Likely pore-forming	[120]
-	Parborlysin/cytotoxin homolog 22 (comp40150/seq2)	*Lineus ruber*	Roscoff, Wimereux, Fra [131]	Likely pore-forming	[120]
-	Parborlysin/cytotoxin homolog 23 (contig21527)	*Lineus sanguineus*	Not stated [131]	Likely pore-forming	[120]
-	Parborlysin/cytotoxin homolog 24 (contig61445)	*Lineus sanguineus*	Not stated [131]	Likely pore-forming	[120]
-	Parborlysin/cytotoxin homolog 25 (contig2073)	*Ramphogordius pseudolacteus ^b^*	Not stated [131]	Likely pore-forming	[120]
-	Parborlysin/cytotoxin homolog 26 (contig6541)	*Ramphogordius pseudolacteus ^b^*	Not stated [131]	Likely pore-forming	[120]
-	Parborlysin/cytotoxin homolog 27 (Locus_8475)	*Riseriellus occultus*	Liverpool, UK [132]	Likely pore-forming	[120]
-	Parborlysin/cytotoxin homolog 28 (Locus_39410)	*Riseriellus occultus*	Liverpool, UK [132]	Likely pore-forming	[120]
-	Parborlysin/cytotoxin homolog 29 (Locus_13571)	*Riseriellus occultus*	Liverpool, UK [132]	Likely pore-forming	[120]
Q76CA2	Echotoxin-2	*Cephalothrix hongkongiensis*	Akkeshi, Hokkaido, Jpn [130]	Pore forming, hemolytic	[124]
Q76CA2	Echotoxin-2	*Cephalothrix linearis*	Not stated	Pore forming, hemolytic	[124]
Q76CA2	Echotoxin-2	*Cerebratulus marginatus*	San Juan Island, WA, USA [130]	Pore forming, hemolytic	[124]
Q76CA2	Echotoxin-2	*Lineus ruber*	Roscoff, Wimereux, Fra [131]	Pore forming, hemolytic	[124]
Q76CA2	Echotoxin-2	*Tubulanus polymorphus*	San Juan Island, WA, USA	Pore forming, hemolytic	[124]
Q66SO3	Galactose-specific lectin nattectin	*Notospermus geniculatus*	Ushimado Mar Inst Okayama Univ, Jpn	Ca^2+^-dependent hemagglutination activity	[15]
A0ZSK3	Neoverrucotoxin subunit alpha	*Cerebratulus marginatus*	San Juan Island, WA, USA [130]	SNTX/VTX toxin; hemolytic activity	[124]
A0ZSK3	Neoverrucotoxin subunit alpha	*Paranemertes peregrina*	San Juan Island, WA, USA	SNTX/VTX toxin; hemolytic activity	[124]
A0ZSK4	Neoverrucotoxin subunit beta ^c^	*Lineus longissimus*	Erdeven + Roscoff, Fra [131]	SNTX/VTX toxin; hemolytic activity	[124]
A0ZSK4	Neoverrucotoxin subunit beta	*Malacobdella grossa*	Rhode Island, USA	SNTX/VTX toxin; hemolytic activity	[124]
Q98989	Stonustoxin	*Lineus lacteus ^a^*	Banyuls, Fréjus, Fra [131]	SNTX/VTX toxin; pore-forming, hemolytic	[124]
Q98989	Stonustoxin	*Lineus ruber*	Roscoff, Wimereux, Fra [131]	SNTX/VTX toxin; pore-forming, hemolytic	[124]
Q91453	Stonustoxin subunit beta	*Tubulanus polymorphus*	San Juan Island, WA, USA	SNTX/VTX toxin; pore-forming, hemolytic	[124]
P58912	Toxin PsTX-60B	*Cephalothrix hongkongiensis*	Akkeshi, Hokkaido, Jpn [130]	MACPF toxin domain; hemolytic	[124]
P58912	Toxin PsTX-60B	*Cephalothrix linearis*	Not stated	MACPF toxin domain; hemolytic	[124]
Q76DT2	Toxin AvTX-60A	*Cephalothrix linearis*	Not stated	MACPF toxin domain; hemolytic	[124]
*Neurotoxins/Acting on ion-channels*					
Q92035	Acetylcholinesterase	*Notospermus geniculatus*	Ushimado Mar Inst Okayama Univ, Jpn	Synaptic cleft hydrolysis of acetylcholine	[15]
P05486	Conophysin-conopressin	*Notospermus geniculatus*	Ushimado Mar Inst Okayama Univ, Jpn	Targets vasopressin-oxytocin related receptors	[15]
6ENA ^d^	Nemertide α-1	*Lineus lacteus ^a^*	Banyuls, Fréjus, Fra [131]	VGSC activator, paralytic	[120]
6ENA ^d^	Nemertide α-1	*Lineus longissimus*	Koster Fiord, Swe	VGSC activator, paralytic	[120]
6ENA ^d^	Nemertide α-1	*Lineus ruber*	Roscoff, Wimereux, Fra [131]	VGSC activator, paralytic	[120]
-	Nemertide α-2	*Lineus longissimus*	Koster Fiord, Swe	Likely VGSC activator, paralytic	[120]
-	Nemertide α-2	*Lineus ruber*	Roscoff, Wimereux, Fra [131]	Likely VGSC activator, paralytic	[120]
-	Nemertide α-3	*Lineus lacteus ^a^*	Banyuls, Fréjus, Fra [131]	Likely VGSC activator, paralytic	[120]
-	Nemertide α-3	*Ramphogordius pseudolacteus ^b^*	Not stated [131]	Likely VGSC activator, paralytic	[120]
-	Nemertide α-4	*Lineus sanguineus*	Not stated [131]	Likely VGSC activator, paralytic	[120]
-	Nemertide α-5	*Ramphogordius pseudolacteus ^b^*	Not stated [131]	Likely VGSC activator, paralytic	[120]
-	Nemertide α-6	*Lineus sanguineus*	Not stated [131]	Likely VGSC activator, paralytic	[120]
-	Nemertide α-7	*Lineus ruber*	Roscoff, Wimereux, Fra [131]	Likely VGSC activator, paralytic	[120]
-	Nemertide α-8	*Riseriellus occultus*	Liverpool, UK [132]	Likely VGSC activator, paralytic; incomplete sequence	[120]
-	Nemertide β-1	*Lineus longissimus*	Koster Fiord, Swe	Neurotoxin B-IV homolog, likely paralytic	[120]
-	Nemertide β-1	*Lineus ruber*	Roscoff, Wimereux, Fra [131]	Neurotoxin B-IV homolog, likely paralytic	[120]
-	Nemertide β-2	*Lineus longissimus*	Koster Fiord, Swe	Neurotoxin B-IV homolog, likely paralytic	[120]
-	Nemertide β-2	*Lineus ruber*	Roscoff, Wimereux, Fra [131]	Neurotoxin B-IV homolog, likely paralytic	[120]
-	Nemertide β-3	*Lineus longissimus*	Koster Fiord, Swe	Neurotoxin B-IV homolog, likely paralytic	[120]
-	Nemertide β-4	*Lineus lacteus ^a^*	Banyuls, Fréjus, Fra [131]	Neurotoxin B-IV homolog, likely paralytic	[120]
-	Nemertide β-5	*Ramphogordius pseudolacteus ^b^*	Not stated [131]	Neurotoxin B-IV homolog, likely paralytic	[120]
-	Nemertide β-6	*Ramphogordius pseudolacteus ^b^*	Not stated [131]	Neurotoxin B-IV homolog, likely paralytic	[120]
-	Nemertide β-7	*Lineus ruber*	Roscoff, Wimereux, Fra [131]	Neurotoxin B-IV homolog, likely paralytic	[120]
-	Nemertide β-8	*Lineus sanguineus*	Not stated [131]	Neurotoxin B-IV homolog, likely paralytic	[120]
-	Nemertide β-9	*Lineus sanguineus*	Not stated [131]	Neurotoxin B-IV homolog, likely paralytic	[120]
Q09GJ9	Cysteine-rich venom protein	*Notospermus geniculatus*	Ushimado Mar Inst Okayama Univ, Jpn	Ca^2+^-channel impairment; paralytic	[15]
Q3SB03	Cysteine-rich venom protein pseudechetoxin-like	*Malacobdella grossa*	Rhode Island, USA	Possible Shk-toxin	[124]
Q3SB03	Cysteine-rich venom protein pseudechetoxin-like	*Paranemertes peregrina*	San Juan Island, WA, USA	Possible Shk-toxin	[124]
Q3SB05	Cys-rich venom protein pseudechetoxin-like	*Notospermus geniculatus*	Ushimado Mar Inst Okayama Univ, Jpn	Possible Shk-toxin	[15]
P69929	Delta-actitoxin-Amc1a	*Notospermus geniculatus*	Ushimado Mar Inst Okayama Univ, Jpn	May inhibit VGSC	[15]
D2Y1Y2	Mu-theraphotoxin-Hhn2a 4	*Notospermus geniculatus*	Ushimado Mar Inst Okayama Univ, Jpn	Blocks TTX-sensitive VGSC	[15]
P0C8G6	Perivitellin-2 67 kDa subunit	*Notospermus geniculatus*	Ushimado Mar Inst Okayama Univ, Jpn	Neurotoxin present in eggs, acts on enterocytes	[15]
P0DKT2	Turripeptide Gsg9.2	*Notospermus geniculatus*	Ushimado Mar Inst Okayama Univ, Jpn	Ion-channel inhibitor	[15]
*Coagulation*					
Q7T3S7	Acidic phospholipase A2 EC-I	*Notospermus geniculatus*	Ushimado Mar Inst Okayama Univ, Jpn	Inhibits platelet aggregation	[15]
Q3C2C1	Phospholipase A2 AP-PLA2-II	*Notospermus geniculatus*	Ushimado Mar Inst Okayama Univ, Jpn	Capillary permeability-increasing and hemorrhagic activities	[15]
Q0ZZJ6	A. superbus venom factor 1	*Notospermus geniculatus*	Ushimado Mar Inst Okayama Univ, Jpn	Complement-activating	[15]
P0CH47	Probable phospholipase A1 magnifin	*Notospermus geniculatus*	Ushimado Mar Inst Okayama Univ, Jpn	Causes platelet aggreagation, phospholipase activity	[15]
E0Y418	Serine protease VLSP-1	*Notospermus geniculatus*	Ushimado Mar Inst Okayama Univ, Jpn	May act in prey hemostasis system	[15]
Q6X5S5	Snaclec 27	*Notospermus geniculatus*	Ushimado Mar Inst Okayama Univ, Jpn	Interferes with platelet aggregation	[15]
Q9DEA1	Snaclec agkicetin-C subunit beta	*Notospermus geniculatus*	Ushimado Mar Inst Okayama Univ, Jpn	Antithrombotic action	[15]
P0C929	Snaclec bothroinsularin subunit alpha	*Notospermus geniculatus*	Ushimado Mar Inst Okayama Univ, Jpn	Inhibits platelet aggregation	[15]
I7ICN3	Snaclec bothroinsularin subunit beta	*Notospermus geniculatus*	Ushimado Mar Inst Okayama Univ, Jpn	Integrin antagonist	[15]
Q38L02	Snaclec dabocetin subunit alpha	*Notospermus geniculatus*	Ushimado Mar Inst Okayama Univ, Jpn	Inhibits platelet aggregation	[15]
Q58L91	Venom prothrombin activator oscutarin-C non-catalytic subunit	*Notospermus geniculatus*	Ushimado Mar Inst Okayama Univ, Jpn	Prothrombin activator; attacks hemostatic system	[15]
J3SBP3	Venom phosphodiesterase 2	*Notospermus geniculatus*	Ushimado Mar Inst Okayama Univ, Jpn	Nuclease activity and platelet aggregator	[15]
*Other proposed activities*					
A0FKN6	Astacin-like metalloprotease toxin 1	*Notospermus geniculatus*	Ushimado Mar Inst Okayama Univ, Jpn	Degrades fibronectin, fibrinogen and gelatin *in vitro*	[15]
P0DN10	U-actitoxin-Avd3i	*Notospermus geniculatus*	Ushimado Mar Inst Okayama Univ, Jpn	Serine protease inhibitor of kallikreins	[15]
P81382	L-amino acid oxidase	*Notospermus geniculatus*	Ushimado Mar Inst Okayama Univ, Jpn	Oxidative deamination of hydrophobic and aromatic residues	[15]
B2DCR8	SE-cephalotoxin	*Cephalothrix linearis*	Not stated	Toxic function unknown	[124]
B2DCR8	SE-cephalotoxin	*Cerebratulus marginatus*	San Juan Island, WA, USA [130]	Toxic function unknown	[124]
B2DCR8	SE-cephalotoxin	*Lineus lacteus ^a^*	Banyuls, Fréjus, Fra [131]	Toxic function unknown	[124]
B2DCR8	SE-cephalotoxin	*Lineus longissimus*	Erdeven + Roscoff, Fra [131]	Toxic function unknown	[124]
B2DCR8	SE-cephalotoxin	*Lineus ruber*	Roscoff, Wimereux, Fra [131]	Toxic function unknown	[124]
P0A968	Cold shock-like protein CspD	*Notospermus geniculatus*	Ushimado Mar Inst Okayama Univ, Jpn	Inhibitor of DNA replication	[15]
P26831	Hyaluronoglycosaminidase	*Notospermus geniculatus*	Ushimado Mar Inst Okayama Univ, Jpn	Likely to act on connective tissue	[15]
P00984	Kunitz-type serine protease inhibitor dendrotoxin E	*Notospermus geniculatus*	Ushimado Mar Inst Okayama Univ, Jpn	Inhibitor of trypsin and chymotrypsin	[15]
Q9KS12	Multifunctional-autoprocessing repeats-in-toxin	*Notospermus geniculatus*	Ushimado Mar Inst Okayama Univ, Jpn	Destroys actin cytoskeleton	[15]
Q66S25	Natterin-1	*Notospermus geniculatus*	Ushimado Mar Inst Okayama Univ, Jpn	Edema and nociception	[15]
Q66S13	Natterin-4	*Lineus lacteus ^a^*	Banyuls, Fréjus, Fra [131]	Edema and nociception	[124]
Q66S13	Natterin-4	*Tubulanus polymorphus*	San Juan Island, WA, USA	Edema and nociception	[124]
Q66S13	Natterin-4	*Notospermus geniculatus*	Ushimado Mar Inst Okayama Univ, Jpn	Edema and nociception	[15]
-	Natterin-4 homolog (Cm6)	*Cerebratulus marginatus*	San Juan Island, WA, USA [130]	Inferred from reciprocal BLAST, likely edema and nociception	[124]
K7Z9Q9	Nematocyst expressed protein 6	*Notospermus geniculatus*	Ushimado Mar Inst Okayama Univ, Jpn	Metalloendopeptidase activity	[15]
A4USB4	Phospholipase D LiSicTox-AlphaVI	*Notospermus geniculatus*	Ushimado Mar Inst Okayama Univ, Jpn	Hydrolysis of sphingomyelin; causes dermonecrosis and inflammation	[15]
Q1W694	Phosopholipase D LiSicTox-betaID1	*Notospermus geniculatus*	Ushimado Mar Inst Okayama Univ, Jpn	Hydrolysis of sphingomyelin; causes dermonecrosis and inflammation	[15]
Q75WF2	Plancitoxin-1	*Cephalothrix hongkongiensis*	Akkeshi, Hokkaido, Jpn [130]	Hepatotoxin	[124]
Q75WF2	Plancitoxin-1	*Notospermus geniculatus*	Ushimado Mar Inst Okayama Univ, Jpn	Hepatotoxin	[15]
Q75WF2	Plancitoxin-1	*Cephalothrix linearis*	Not stated	Hepatotoxin	[124]
Q75WF2	Plancitoxin-1	*Cerebratulus marginatus*	San Juan Island, WA, USA [130]	Hepatotoxin	[124]
Q75WF2	Plancitoxin-1	*Lineus lacteus ^a^*	Banyuls, Fréjus, Fra [131]	Hepatotoxin	[124]
Q75WF2	Plancitoxin-1	*Lineus longissimus*	Erdeven + Roscoff, Fra [131]	Hepatotoxin	[124]
Q75WF2	Plancitoxin-1	*Lineus ruber*	Roscoff, Bretagne, Wimereux, France [131]	Hepatotoxin	[124]
Q75WF2	Plancitoxin-1	*Malacobdella grossa*	Rhode Island, USA	Hepatotoxin	[124]
Q75WF2	Plancitoxin-1	*Paranemertes peregrina*	San Juan Island, WA, USA	Hepatotoxin	[124]
Q75WF2	Plancitoxin-1	*Tubulanus polymorphus*	San Juan Island, WA, USA	Hepatotoxin	[124]
C1IC50	Protease inhibitor-1	*Notospermus geniculatus*	Ushimado Mar Inst Okayama Univ, Jpn	Serine protease inhibitor	[15]
A8YPR9	Snake venom metalloprotease inhibitor 02A10	*Notospermus geniculatus*	Ushimado Mar Inst Okayama Univ, Jpn	Metalloproteinase inhibitor during glandular storage	[15]
P86548	Soybean toxin 17 kDa chain	*Notospermus geniculatus*	Ushimado Mar Inst Okayama Univ, Jpn	Involved in plant defense	[15]
B5U2W0	Venom serine protease Bi-VSP	*Notospermus geniculatus*	Ushimado Mar Inst Okayama Univ, Jpn	Serine protease, induces melanization in target insects	[15]

ID is short for UniProtKB entry (uniprot.org). The proposed functions listed are the result of automatic annotation, extracted from uniprot.org entries, or for sequences lacking uniprot entry, inferred from activities of known homologs by the authors of this review. ^a^ In source also referred to as *Ramphogordius lacteus* (synonymous). ^b^ In source referred to as *Lineus pseudolacteus* (synonymous). ^c^ In source assigned as subunit alpha. ^d^ PDB code. Species are denoted according to WoRMS, World Register of Marine Species as of 19-02-11 [54].
